# The Formin Fmn2b Is Required for the Development of an Excitatory Interneuron Module in the Zebrafish Acoustic Startle Circuit

**DOI:** 10.1523/ENEURO.0329-20.2021

**Published:** 2021-07-08

**Authors:** Dhriti Nagar, Tomin K. James, Ratnakar Mishra, Shrobona Guha, Shawn M. Burgess, Aurnab Ghose

**Affiliations:** 1 Indian Institute of Science Education and Research (IISER) Pune, Pune 411008, India; 2 Translational and Functional Genomics Branch, National Human Genome Research Institute, National Institutes of Health, Bethesda, MD 20892

**Keywords:** acoustic startle, Fmn2, Mauthner cell, neurodevelopment, spiral fiber neuron, zebrafish

## Abstract

The formin family member Fmn2 is a neuronally enriched cytoskeletal remodeling protein conserved across vertebrates. Recent studies have implicated Fmn2 in neurodevelopmental disorders, including sensory processing dysfunction and intellectual disability in humans. Cellular characterization of Fmn2 in primary neuronal cultures has identified its function in the regulation of cell-substrate adhesion and consequently growth cone translocation. However, the role of Fmn2 in the development of neural circuits *in vivo*, and its impact on associated behaviors have not been tested. Using automated analysis of behavior and systematic investigation of the associated circuitry, we uncover the role of Fmn2b in zebrafish neural circuit development. As reported in other vertebrates, the zebrafish ortholog of Fmn2 is also enriched in the developing zebrafish nervous system. We find that Fmn2b is required for the development of an excitatory interneuron pathway, the spiral fiber neuron, which is an essential circuit component in the regulation of the Mauthner cell (M-cell)-mediated acoustic startle response. Consistent with the loss of the spiral fiber neurons tracts, high-speed video recording revealed a reduction in the short latency escape events while responsiveness to the stimuli was unaffected. Taken together, this study provides evidence for a circuit-specific requirement of Fmn2b in eliciting an essential behavior in zebrafish. Our findings underscore the importance of Fmn2 in neural development across vertebrate lineages and highlight zebrafish models in understanding neurodevelopmental disorders.

## Significance Statement

Fmn2 is a neuronally enriched cytoskeletal remodeling protein linked to neurodevelopment and cognitive disorders in humans. Recent reports have characterized its function in growth cone motility and chemotaxis in cultured primary neurons. However, the role of Fmn2 in the development of neural circuits *in vivo* and its implications in associated behaviors remain unexplored. This study shows that Fmn2b is required for the development of neuronal processes in the acoustic startle circuit to ensure robust escape responses to aversive stimuli in zebrafish. Our study underscores the crucial role of the non-diaphanous formin, Fmn2b, in establishing neuronal connectivity.

## Introduction

The behavioral repertoire of an organism is contingent on accurate neural connectivity of the nervous system. During development, individual neurites are guided to reach their predetermined targets by environmental cues. Specialized tips of growing neurites, the growth cones, employ a plethora of cytoskeleton regulatory proteins to locally remodel the cytoskeleton and execute directional translocation in response to guidance cues (Lowery and Van Vactor, 2009). Precisely regulated, dynamic remodeling of the actin and microtubule cytoskeletons enables the growth cone to navigate toward its synaptic target accurately. Actin-rich growth cone filopodia express guidance receptors and function as chemosensory antennae. Differential stabilization of filopodia, via microtubule capture, allows the microtubule network to advance in a spatially biased manner to bring about directional forward displacement (Dent et al., 2011). Further, the cytoskeleton modulates the adhesion of the growth cone to the surrounding matrix and the ability to generate traction forces (Kerstein et al., 2015). There are several classes of proteins involved in active cytoskeleton remodeling. One such family of proteins is the formin family of proteins. Formins are a conserved family of actin nucleators and processive elongators of non-branched actin filaments characterized by the FH1 and FH2 domains (Higgs, 2005; Higgs and Peterson, 2005; Breitsprecher and Goode, 2013). Members of the formin family are known to regulate other aspects of cytoskeleton dynamics, like F-actin bundling, actin-microtubule cross talk, and the adhesion of the growth cone to the extracellular matrix (Kawabata Galbraith and Kengaku, 2019).

Most formins are broadly expressed in multiple tissues types (Schönichen and Geyer, 2010; Breitsprecher and Goode, 2013; Krainer et al., 2013; Dutta and Maiti, 2015; Kawabata Galbraith and Kengaku, 2019) but the non-diaphanous-related formin, Formin-2 (Fmn2), is found to be enriched in the developing and the mature nervous systems of mice, humans, and chicken (Leader and Leder, 2000; Katoh and Katoh, 2004; Dutta and Maiti, 2015; Sahasrabudhe et al., 2016). Fmn2 has been implicated in neurodevelopmental disorders, intellectual disability, age-related dementia, microcephaly and sensory processing dysfunction in humans (Perrone et al., 2012; Almuqbil et al., 2013; Law et al., 2014; Agís‐Balboa et al., 2017; Anazi et al., 2017; Marco et al., 2018). Recent studies identify the involvement of Fmn2 in regulating of spine density in hippocampal neurons (Law et al., 2014) and in the development of the corpus callosum in humans (Gorukmez et al., 2020).

So far, there is little known about the mechanisms by which Fmn2 causes neurodevelopmental defects. Fmn2 is required for growth cone motility and axonal pathfinding of spinal neurons. Filopodial stability and the dynamics of the adhesion complexes between the growth cone and the substrate are regulated by Fmn2 (Sahasrabudhe et al., 2016; Ghate et al., 2020). Another study has identified actin-microtubule cross talk mediated by Fmn2 as a mechanism underlying growth cone turning (Kundu et al., 2021). These reports primarily use cultured primary neurons which precludes investigating gene function in assembling neural circuits giving rise to appropriate behaviors.

Zebrafish is an excellent vertebrate model to interrogate gene function in the establishment of neural circuits *in vivo* and associated behaviors (Kalueff et al., 2013; Mcarthur et al., 2020). One of the most studied neural circuits is the Mauthner cell (M-cell)-mediated acoustic startle circuit (Swain et al., 1993; Jontes et al., 2000; Korn and Faber, 2005; Burgess et al., 2009; Sillar, 2009; Issa et al., 2011; Kinkhabwala et al., 2011; Hale et al., 2016; Liu and Hale, 2017; Hecker et al., 2020). Acoustic and tactile stimuli from the environment are processed by the M-cells aided by regulatory excitatory and inhibitory interneuron module and relayed to the motor neurons downstream (Korn and Faber, 2005). Mauthner-mediated escapes are essential to the survival of the zebrafish in the wild environment. The acoustic startle circuit comprising of sensory inputs to the M-cells and regulatory interneurons imparts the ability to respond in time to evade a predator.

In this study, we show that *fmn2b* expression is enriched in the zebrafish nervous system and is necessary for the development of an excitatory interneuron pathway indispensable for M-cell-mediated fast escape responses to aversive stimuli.

## Materials and Methods

### Zebrafish maintenance

Locally sourced wild-type strain of zebrafish, bred in the lab for three generations were used in all the experiments. Adult zebrafish were raised in a recirculating aquarium system (Techniplast) at 28.5°C under a 14/10 h light/dark cycle. Embryos were collected and raised in E3 buffer (5 mm NaCl, 0.33 mm MgSO_4_, 0.17 mm KCl, 0.33 mm CaCl_2_, and 5% methylene blue) at 28.5°C and used at different stages as previously described (Kimmel et al., 1995). The following transgenic zebrafish lines were used: Tg(*cldn*-b:Lyn-GFP) (Haas and Gilmour, 2006) and Tg(*brn3c*:GAP43-GFP) (Xiao et al., 2005).

### Whole-mount *in situ* hybridization

Total RNA was isolated from 48 h postfertilization (hpf) wild-type embryos using the RNeasy mini kit (QIAGEN) and reverse transcribed using the SuperScript IV RT kit (ThermoFisher) to obtain cDNA. The cDNA was amplified for a 366-bp-long gene-specific region of *fmn2b* corresponding the 5′ UTR and exon 1 flanked with T7 and T3 promoter sequences in the antisense and sense direction, respectively.

Primer sequences: Fmn2b_ISH_5UTR_F_T3, GCAATTAACCCTCACTAAAGGGATGCGTTGTTGTGTTTGTG; and Fmn2b_ISH_5UTR_R_T7, TAATACGACTCACTATAGGGGCTCTCGCTGTCTGATGAAG.

The amplified product was purified and used as the template for *in vitro* transcription of antisense and sense probes against *fmn2b* mRNA. Zebrafish embryos ranging from one cell stage to 96 hpf were used for whole-mount *in situ* hybridization experiments as described previously (Thisse and Thisse, 2008). BM Purple was used as a chromogenic substrate for detection.

### Morpholino, Cas9-sgRNA, and mRNA injections

Two morpholinos targeting *fmn2b* were obtained from Gene Tools. The splice blocking morpholino targets the intron between exons 5 and 6 and ensures the inclusion of a stop codon in the translation frame. The translation blocking morpholino binds early on in the first exon. The control morpholino targets the β-thalassemia causing mutation in the human β-globin gene. The control morpholino has no reported off-target effects and is used as a negative control.

The sequences of the morpholinos used are the following:

MO control: 5′-CCTCTTACCTCAGTTACAATTTATA-3′;

MO SB fmn2b: 5′-ACAGAAGCGGTCATTACTTTTTGGT-3′; and

MO TB fmn2b: 5′-ATGAGCGGCGGCGGTTTCAAGCCAT-3′.

All experiments were done by injecting 2-nl volume of the MO control and MO SB fmn2b (1 ng/nl; 2 ng/embryo) in the cytoplasm of the one-cell stage embryos. For MO TB fmn2b dose dependence, 4 and 8 ng/embryo doses of the morpholino were injected in the yolk of embryos in addition to the cytoplasmic injection of 2 ng/embryo. The injection volume was calibrated at 2 nl/embryo for each needle before injection. After injections, embryos were washed and raised in E3 buffer (supplemented with methylene blue) at 28.5°C until the desired developmental stage with regular cleaning. For immunostaining and live imaging experiments, the buffer was supplemented with 0.003% phenylthiourea (PTU; Sigma) to remove pigmentation from the skin. For rescue experiments, capped mRNA was synthesized using the SP6 mMessage mMachine RNA synthesis kit (Ambion) from pCS2-mFmn2-GFP plasmid (provided by Prof. Philip Leder, Harvard Medical School). After purification using RNeasy MinElute Cleanup kit (QIAGEN), 300 pg of mFmn2-GFP capped RNA was co-injected with MO SB Fmn2b morpholino per embryo.

Two sgRNAs targeting exon 1 of *fmn2b* were designed as previously described (Varshney et al., 2016). sgRNAs were designed as oligonucleotides with a T7 promoter upstream and annealed to obtain DNA template for *in vitro* transcription using T7 HiScribe kit (NEB). Cas9 mRNA was synthesized using the T3 mMessage mMachine RNA synthesis kit (Ambion) from pT3TS-nCas9n plasmid (provided by Shawn Burgess, NHGRI, NIH). After purification of the sgRNAs and Cas9 mRNA, 300 pg of Cas9 mRNA was injected as control. To generate crispants, 30 pg or 100 pg of both sgRNAs were injected along with the Cas9 mRNA. Sequences for the sgRNAs are the following:

sgRNA-1: 5′-GGGCGAGAGGCCTCGGCTGG-3′ and

sgRNA-2: 5′- GCGGATCCTCCCTCTGCATG-3′.

We injected Cas9 mRNA with or without the two sgRNAs (sgRNA-1 and sgRNA-2 together) at the one-cell stage. To ascertain the activity of the sgRNAs, we extracted the genomic DNA from 24-hpf crispants using a modified HotSHOT protocol for zebrafish (Meeker et al., 2007). The genomic DNA from the Cas9 mRNA only control (*n* = 8) and the crispants (*n* = 15) was used for amplifying the region flanking each of the sgRNA loci by PCR and Sanger sequencing was performed on the amplicons. We observed mutations in all the crispant embryos that were sequenced. Out of the 15 embryos, 93.3% crispants showed indels at the sgRNA-1 locus and 53.3% crispants showed indels at the sgRNA-2 locus. The indels are summarized in Extended Data Figure 5-2. Furthermore, all the remaining crispants without indels also showed base changes with some of them causing premature stop codons to occur. The Cas9 mRNA only control showed no indels or base changes at both the sgRNA loci. This suggests that the two sgRNAs injected in the crispants cause indels or premature stop codons in a substantial fraction of the population potentially causing frameshift mutations and therefore, a truncated protein.

Primer sequences for sanger sequencing of genomic DNA amplicons from crispants are as follows: Fmn2b_PCR_F_sgRNA1, AAGCGTAAGAACCAGAATAAGC; Fmn2b_PCR_R_sgRNA1, TCATCCGAATGGCTTGC; Fmn2b_PCR_F_sgRNA2, GAGTGTGCAGGAAGATGC; and Fmn2b_PCR_R_sgRNA2, GTGACGAAGGAGAGGTACAG.

### Validation of splice blocking morpholino

The spice blocking morpholino MO SB fmn2b was validated by RT-PCR. Total RNA isolated from morpholino injected 48-hpf embryos (RNeasy mini kit, QIAGEN) was reverse transcribed using the SuperScript IV RT kit (ThermoFisher). The cDNA obtained was amplified by the following primers flanking the intron between exon 5 and exon 6. Primer sequences: MOSBFmn2b_RT_FWD: 5′-TCTGTTTGCATTGGGAGC-3′ and MOSBFmn2b_RT_REV: 5′-CTTGGTCTTTGACCTGCTGAT-3′.

In control morphants, the expected amplicon size is 251 bp, whereas in Fmn2b morphants, the amplicon size was expected and found to be 550 bp because of blocked splicing of the intron between exons 5 and 6.

### Whole-mount immunostaining, FM-4-64 labeling, tetramethylrhodamine (TMR) dextran labeling, and fluorescence microscopy

PTU-treated embryos were collected at desired stages and fixed in 4% formaldehyde overnight at 4°C. The fixed embryos were stained as described earlier (Hatta, 1992) using 3A10 (DSHB; 1:50) antibody in blocking buffer. The larvae were washed with PBS-Triton X-100 (0.5%) followed by blocking in 5% BSA. For RMO-44 immunostaining, embryos at the desired stage were fixed using 2% trichloroacetic acid (TCA) at room temperature for 3 h, washed with PBS followed by acetone permeabilization for 30 min at −20°C. The embryos were then washed with distilled water, incubated in blocking buffer for 1 h and transferred to RMO-44 (1:100) and incubated at room temperature for 12 h. The larvae were then stained with anti-mouse Alexa Fluor 568/488 (1:200) overnight at 4°C in blocking buffer. After extensive washing with PBS-Triton X-100 (0.5%), the embryos were cleared in 50% glycerol and mounted dorsal side down on a glass bottom Petri dish using low gelling agarose (Sigma). To label the hair cells of the inner ear cristae, 1 nl solution of 3 μm FM-4-64 (Invitrogen) dissolved in DMSO was injected in the otic cavity of 96-hpf zebrafish embryos mounted laterally in low gelling agarose (Sigma). The injected embryos were removed from the gel using E3 buffer and imaged within 1 h of injections.

Retrograde labeling of the reticulospinal neurons was done using TMR dextran (3000 MW) by microinjection of three pulses of 1 nl each in the ventral side of the spinal cord of 3-days post fertilization (dpf) zebrafish larvae mounted in 1% low gelling agarose. The injected larvae were allowed to recover in E3 medium overnight at 28°C and fixed with 4% PFA at 4 dpf for 3 h at room temperature. The fixed larvae were cleared using 50% glycerol and mounted in a coverslip sandwich for imaging. The samples were imaged on the LSM 780 confocal microscope (Zeiss) using a 25× oil immersion objective (NA 1.4).

### Behavior experiment set up and behavior analysis

We designed a behavioral assay as described previously (Lacoste et al., 2015) to screen larvae based on their response to a mechano-acoustic stimulus. The control and Fmn2b morphants were anaesthetized using 0.03% tricaine (MS-222, Sigma), head-restrained using 1% low gelling agarose and their tails were suspended in E3 buffer, in a 35-mm Petri dish. All the fish were habituated for 30 min in the behavior room maintained at a temperature of 27°C. Individual dishes containing one larva were placed and taped onto the behavioral setup, as shown in Figure 2*A*. Up to six taps were delivered using a 14 V DC solenoid (obtained from a local store) to the dish at an interval of 10 s at an intensity corresponding to 14 V from a power supply. The setup comprises of an automated stimulus delivery control unit (Arduino), a solenoid with a piston driven by a variable power supply, a piezo sensor (SparkFun) to detect the stimulus, and a feedback TTL pulse to the camera to mark the reception of the stimulus. This allowed precise marking of the stimulus delivery directly onto the images acquired using a high-speed video camera (AVT Pike, F-032B) at 640 fps. The secure image signature (SIS) feature of the camera was used to put a time-stamp on individual frames acquired. The recordings were analyzed using a custom-written Python program to extract the time-stamp, time of stimulus delivery and skeletonizing the fish to obtain coordinates of a spline curve fit to the fish shape in every frame. The skeleton was segmented into 20 points, and the last five points were used for further calculations. The program calculates the tail angle from last five points on the fish skeleton with respect to the restrained head segment. An event qualifies as an escape response if the angle crosses a threshold of 60° from the rest position (Lacoste et al., 2015).

The following parameters have been quantified, as mentioned below:

Latency to first movement: time taken by the fish to initiate movement poststimulus delivery, marked by an angle change greater than 5°.

C-bend max: maximum angle of C-bend escape (with respect to the head restrained segment).

Latency to C-bend max: time taken by the fish to reach the maximum C-bend escape angle, calculated by subtracting latency to first movement from the total time taken to reach maximum angle.

### Figures and statistical analysis

All the analysis was performed in a genotype blinded manner. The images were processed in Fiji and assembled as figure panels using Inkscape. The data for all measurements are represented as (median; [95% confidence intervals; 95%CIs]; number of events) for Figures 2 and 3 in the results section. Data obtained from various experiments were analyzed using an estimation statistics approach (Ho et al., 2019) and the median difference values and respective permutation test *p* values are indicated in the figure legends. All data points have been presented as a swarm plot for individual values displaying the underlying distribution. The effect size is presented as a bootstrap 95%CI below the swarm plots showing the median differences obtained by resampling the data 5000 times. The plots were generated using the web application available at https://www.estimationstats.com/#/.

χ^2^ test was performed on the contingency tables for the data represented in Figure 5*D*,*H* using GraphPad Prism 8.

### Ethics approval

All protocols used in this study were approved by the Institutional Animal Ethics Committee and the Institutional Biosafety Committee of Indian Institute of Science Education and Research (IISER) Pune.

### Availability of data and material

All data generated or analyzed during this study are included in this published article. The raw data are available from the corresponding author on reasonable request.

## Results

### Fmn2b, the zebrafish ortholog of Fmn2, is expressed in the nervous system

**Figure 1.**
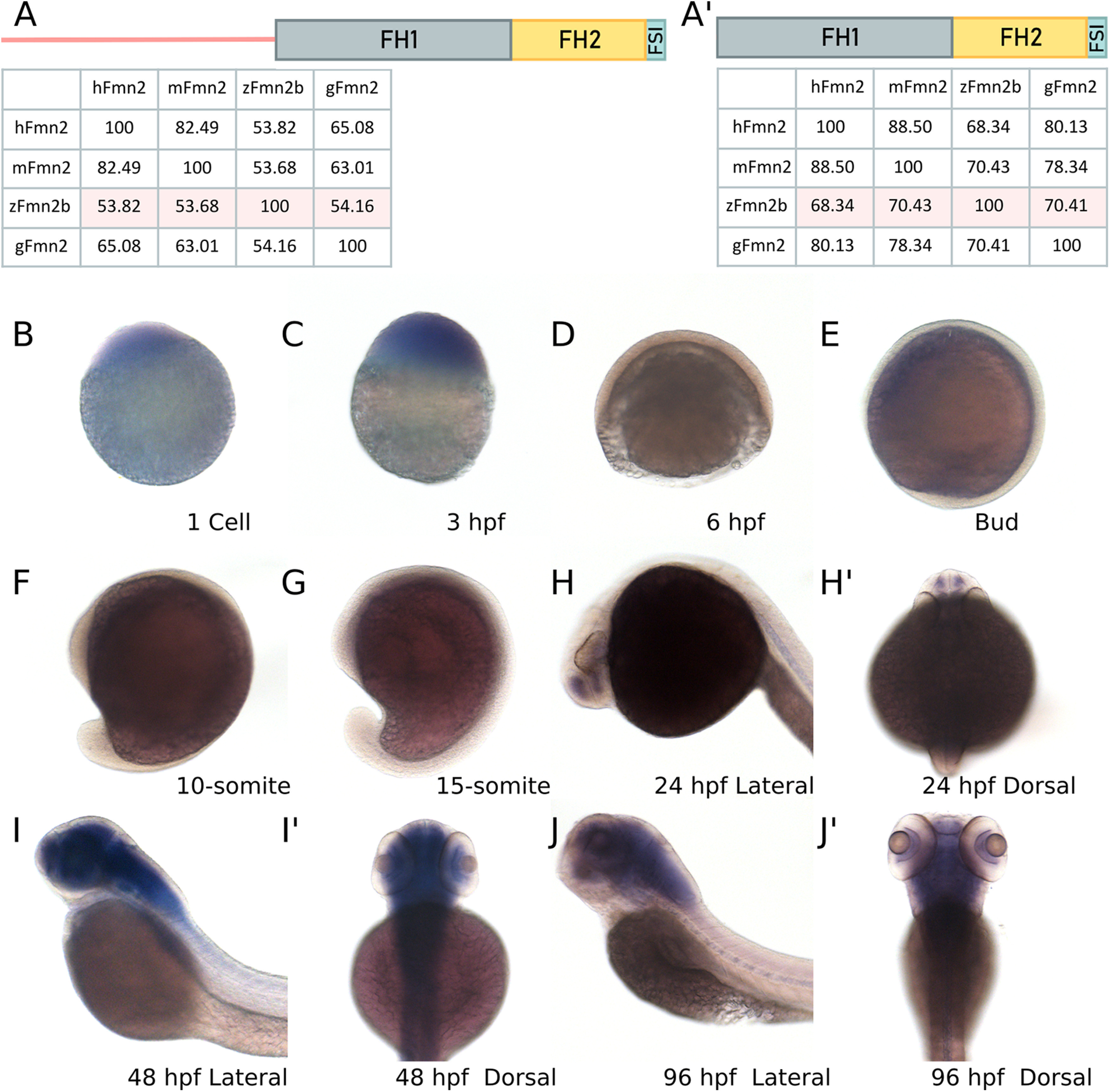
*fmn2b* mRNA is maternally deposited and enriched in the zebrafish nervous system. Amino acid sequence alignments were obtained using EMBL Clustal Omega for human Fmn2 (hFmn2), mouse Fmn2 (mFmn2), zebrafish Fmn2b (zFmn2), and chicken Fmn2 (gFmn2) to compare conservation of sequence across vertebrate species. Percent identity matrices are shown for comparison of full length Fmn2 and the functional domains of Fmn2 across species. ***A***, Full-length zFmn2 showed 53.82%, 53.68%, and 54.16% sequence similarity for human, mouse, and chicken Fmn2, respectively. ***A’***, On comparing the functional domains of Fmn2, namely FH1, FH2, and FSI domain, we found 68.34%, 70.43%, and 70.41% sequence similarity with human, mouse, and chicken Fmn2, respectively. ***B–J***, Representative images of whole-mount mRNA *in situ* hybridization showing the *fmn2b* mRNA expression pattern in the developing zebrafish embryo. *fmn2b* mRNA can be found in (***B***) one cell stage and (***C***) 3-hpf embryos suggesting maternal deposition. The mRNA expression is negligible during (***D***) 6 hpf, (***E***) bud stage, (***F***) 10-somite, and (***G***) 15-somite embryos. *fmn2b* mRNA expression is regained in (***H***) 24-hpf embryos and continues to be expressed until 96 hpf (***I–J’***). hpf, hours post fertilization.

Using reciprocal BLASTp, we found that Fmn2b (E7F517; UNIPROT) has the highest sequence identity with human Fmn2 (53.82%; Fig. 1*A*). The next best hit, Fmn2a (X1WC43; UNIPROT), was found to have considerably less sequence identity with human Fmn2 (44.75%). While Fmn2a and has a characteristic FH2 domain, the FH1 domain is truncated to 33 aa. Further, mRNA corresponding to fmn2a was expressed at very low levels in zebrafish larvae with no detectable signal in the nervous system up to 96 hpf (data not shown). Therefore, we further characterized the role of Fmn2b (E7F517) in the developing nervous system of zebrafish.

High amino acid sequence similarity was observed between the human (Q9NZ56), mouse (Q9JL04), chick (NP_001317992) and zebrafish Fmn2 (E7F517) orthologs with the C-terminal FH1, FH2, and Formin spire interaction (FSI) domains showing ∼70% similarity (Fig. 1*A*,*A’*). Using whole-mount *in situ* hybridization, we evaluated the expression pattern of *fmn2b* mRNA in zebrafish across developmental stages (Fig. 1*B–J*). We found that *fmn2b* mRNA is maternally deposited, as seen in one-cell stage zebrafish embryos, and the expression persists until 3 hpf. There was no discernible *fmn2b* mRNA expression at stages from 6 to 18 hpf. The mRNA expression reappeared in the telencephalon/forebrain at 24 hpf (Fig. 1*H*). At stages 48 hpf onwards, the expression extends to the diencephalon/midbrain, the rhombencephalon/hindbrain, the spinal cord and the retinal ganglion cells (RGCs) layer of the eye. Similar expression pattern is observed at 96 hpf (Fig. 1*I*,*J*).

In line with previous studies showing the expression of *fmn2* in the nervous system of human, mice (Leader and Leder, 2000), and chick (Sahasrabudhe et al., 2016), *fmn2* mRNA is also enriched in the zebrafish nervous system.

### Fmn2b morphants exhibit a delay in initiating the escape response

To test the contribution of Fmn2b in development of the nervous system, we used a morpholino-mediated knock-down approach. We designed two *fmn2b* specific antisense morpholinos, one translation blocking (MO TB Fmn2b) and one splice blocking (MO SB Fmn2b). The MO TB Fmn2b morpholino targets the first exon, and the MO SB Fmn2b blocks splicing at the boundary of the fifth exon and intron (Extended Data Fig. 2-1*A*). To test the extent of knock-down by MO SB Fmn2b, RT-PCR was performed to ensure incorporation of the intron after exon 5, causing inclusion of a stop codon in the translation frame. In 48 hpf MO SB Fmn2b morphants, the majority of the *fmn2b* mRNA was present in the splice-blocked form evident from the 550-bp amplicon corresponding to the inclusion of the intron between exons 5 and 6, as compared with the spliced version with an expected amplicon size of 251 bp (Extended Data Fig. 2-1*B*).

Out of all the embryos injected at one-cell stage with MO TB Fmn2b and MO SB Fmn2b, around 40% embryos exhibited morphologic defects, like microcephaly, cardiac edema and axial curvature (Extended Data Fig. 2-1*E*). These were excluded from additional experiments. The larvae with intact otolith, inflated swim bladders and body length (6.5–7 mm) comparable to the control morphants were used for all analyses to ensure unbiased observations in locomotor behavior assessment. The parameters used for classification of morphologically defective and normal embryos are summarized in Extended Data Figure 2-1*F*.

**Figure 2.**
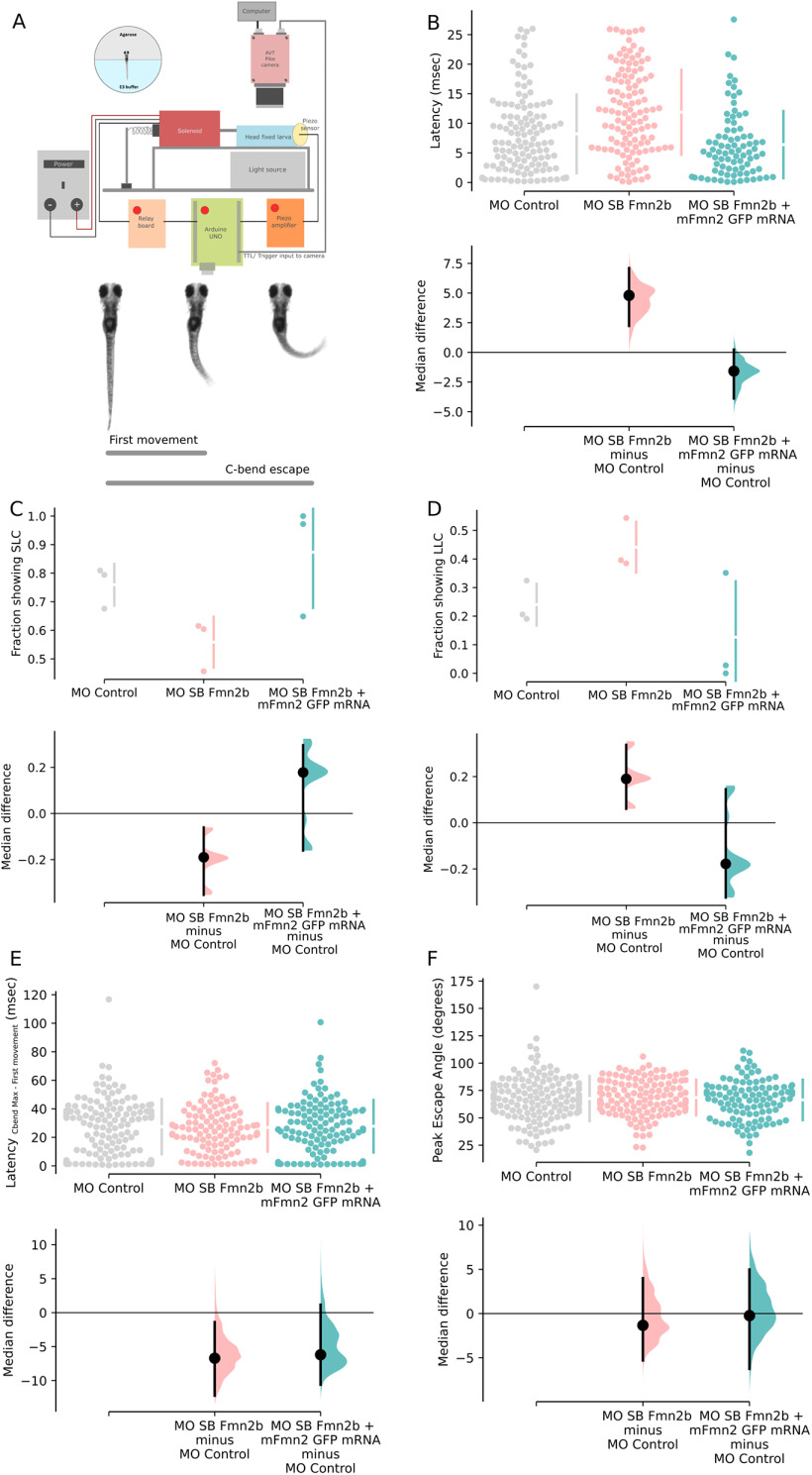
Fmn2b knock-down increased the latency to respond to mechano-acoustic stimulus. Fmn2b knock-down using translation and splice blocking morpholinos (morpholino validation in Extended Data Fig. 2-1*A*,*B*) results in deficits in latency to acoustic startle response. Fmn2b morphants without any morphologic defects were used for the following experiments. The morphologic defects are summarized in Extended Data Figure 2-1*E*,*F*. Fmn2b morphants do not show any changes in the responsiveness to the acoustic stimuli (Extended Data Fig. 2-1*C*). ***A***, Schematic of the automated stimulus delivery apparatus and description of movements qualifying as the first movement for latency calculation and maximum C-bend for the escape response following the mechano-acoustic stimulus. ***B–F***, The median difference comparisons against the control morpholino (MO control) are shown in the Cumming estimation plots. The raw data are plotted on the upper axes as individual dots. The vertical gapped lines summarize the median ± SD for each group. On the lower axes, median differences are plotted as bootstrap sampling distributions obtained by resampling the data 5000 times. The median difference is depicted as a dot for respective groups as compared with the shared control. The 95%CI is indicated by the ends of the vertical error bars. ***B***, Latency to first movement (ms) is plotted in this graph. The unpaired median difference between MO control (*n* = 126) and MO SB Fmn2b (*n* = 120) is 4.8 [95.0%CI 2.23, 7.1]. The *p* value of the two-sided permutation *t* test on the median differences is 0.0. The unpaired median difference between MO control and MO SB Fmn2b + mFmn2-GFP mRNA (*n* = 83) is −1.58 [95.0%CI −3.89, 0.232]. The *p* value of the two-sided permutation *t* test on the median differences is 0.081. The latency to first movement is decreased in Fmn2b morphants injected with the translation blocking morpholino MO TB Fmn2b as compared with the control morphants (Extended Data Fig. 2-1*D*). ***C***, Values calculated for the fraction of the population (*n* = 3) showing SLC response (<13 ms) are plotted in this graph. The unpaired median difference between MO control and MO SB Fmn2b is −0.201 [95.0%CI −0.308, −0.107]. The *p* value of the two-sided permutation *t* test is 0.0. The unpaired median difference between MO control and MO SB Fmn2b + mFmn2-GFP mRNA is 0.114 [95.0%CI −0.151, 0.266]. The *p* value of the two-sided permutation *t* test is 0.357. ***D***, Values calculated for the fraction of the population (*n* = 3) showing LLC response (13–26 ms) are plotted in this graph. The unpaired median difference between MO control and MO SB Fmn2b is 0.201 [95.0%CI 0.104, 0.303]. The *p* value of the two-sided permutation *t* test is 0.0. The unpaired median difference between MO control and MO SB Fmn2b + mFmn2-GFP mRNA is −0.114 [95.0%CI −0.276, 0.111]. The *p* value of the two-sided permutation *t* test is 0.29. ***E***, The difference of time taken to achieve the maximum C-bend angle and the latency to first movement for each trial is plotted in this graph. The unpaired median difference between MO control and MO SB Fmn2b is −6.71 [95.0%CI −12.2, −1.4]. The *p* value of the two-sided permutation *t* test is 0.0526. The unpaired median difference between MO control and Rescue is −6.2 [95.0%CI −10.6, 1.17]. The *p* value of the two-sided permutation *t* test is 0.152. ***F***, This graph shows the maximum angle (C-bend max) attained during the escape response. The unpaired median difference between MO control and MO SB Fmn2b is −1.34 [95.0%CI −5.31, 4.0]. The *p* value of the two-sided permutation *t* test is 0.694. The unpaired median difference between MO control and rescue is −0.235 [95.0%CI −6.26, 4.98]. The *p* value of the two-sided permutation *t* test is 0.875. For all the estimation statistics analysis, the effect sizes and CIs are reported as effect size (CI width lower bound; upper bound). SLC: Short latency C-bend escapes, LLC: Long latency C-bend escapes.

To evaluate the effect of Fmn2b knock-down on behavior, we recorded the responses of control and Fmn2b morphants to manual tapping of the dish containing the larvae. In this preliminary experiment, we observed uncoordinated locomotion in Fmn2b morphants. To better resolve these behavioral defects, we employed high-speed video recording and tested the response of morphants to mechano-acoustic stimuli. In this assay, we subjected 96-hpf head restrained morphants to a tap on the dish in which they were housed and measured their response by tracking the movement of their tails. The response of the morphants was recorded at 640 fps using a high-speed video recording camera (AVT Pike, F-032B). The taps were delivered at 10-s intervals, controlled by an Arduino UNO microprocessor (Fig. 2*A*). The recorded videos were analyzed using a custom written python program to extract time-stamp, and the body axis coordinates after skeletonizing the shape of the fish (Movie 1).

10.1523/ENEURO.0329-20.2021.f2-1 Extended Data Figure 2-1 Morpholino design, validation, and assessment of morphological defects in morphants. ***A***, Schematic showing target regions for the two morpholinos used in the experiments. MO TB Fmn2b targets exon 1 to block translation. MO SB Fmn2b targets the exon 5–intron 5 boundary to cause retention of intron 5 between exons 5 and 6, leading to the occurrence of a premature stop codon. Both morpholinos ensure that the functional domains are not translated in Fmn2b morphants. ***B***, Validation of knock-down by MO SB Fmn2b morpholino was done using RT-PCR on cDNA obtained from MO control and MO SB Fmn2b-injected embryos. The amplification of a 550-bp amplicon from MO SB Fmn2b morphants cDNA corresponds to inclusion of intron 5 because of efficient splice blocking by the morpholino. ***C***, Responsiveness is quantified as the percentage of larvae responding to acoustic stimuli in MO control (95.2%), MO TB Fmn2b (92.9%), and MO SB Fmn2b (94%)-injected embryos. ***D***, Behavioral analysis of MO TB Fmn2b morphants is summarized in the Cumming plot. MO TB Fmn2b morphants also exhibit increased latency defect. The unpaired median difference between MO control and MO TB Fmn2b is 4.2 [95.0%CI 2.0, 6.3]. The *p* value of the two-sided permutation *t* test is 0.0004. The effect sizes and CIs are reported as effect size [CI width lower bound; upper bound]. ***E***, Graph summarizing morphological defects in Fmn2b morphants injected with 2 ng of MO SB Fmn2b. The percentage of embryos showing the various defects are indicated at the bottom of each bar corresponding to the defect. A total of 40.04% embryos in the morphant population showed defects spanning from a few or all of the defects listed. Each bar represents the percentage of embryos exhibiting that morphological defect. Morphologically aberrant embryos often exhibited multiple defects together. ***F***, Table enlisting the parameters used to distinguish between morphologically aberrant and normal embryos. These parameters were used for classification of both morphant and crispant populations. Download Figure 2-1, TIF file.

Movie 1. Tracking the escape response in a 96-hpf wild-type zebrafish larva. The movie shows a wildtype larva being tracked by a custom written code which extracts time-stamp from the SIS on the frames and skeletonizes the fish to obtain coordinates for further calculations. 10.1523/ENEURO.0329-20.2021.video.1

The latency to first movement, the maximum escape angle (C-bend Max) and the latency to achieve maximum angle in an escape response (latency_C-bend max – first movement_) were calculated.

The tap strength using the piston was controlled by varying the input voltage on the variable power supply. For all the experiments, tap strength corresponding to 13 V was used. This tap strength was optimized to achieve ∼95% responsiveness in the control zebrafish larvae. The responsiveness of the Fmn2b morphants (injected with 2 ng/embryo MO SB Fmn2b) to the same mechano-acoustic stimulus was comparable (94%) to the control animals (Extended Data Fig. 2-1*C*). On the other hand, we observed that Fmn2b morphants showed an increased latency to first movement (11.54 ms; [9.36,13.38]; *n* = 120; Fig. 2*B*) in response to the mechano-acoustic stimulus as compared with the control morphants (6.74 ms; [5.39,9.06]; *n* = 126; Fig. 2*B*). To ensure that the morpholino’s effect was specific to Fmn2b, we co-injected mouse Fmn2-GFP (mFmn2-GFP) mRNA, which is resistant to the anti-zebrafish Fmn2b morpholino, along with the Fmn2b morpholinos. The increased latency could be rescued in Fmn2b morphant larvae co-injected with mFmn2-GFP mRNA (5.16 ms; [4.03,6.29]; *n* = 83; Fig. 2*B*). Similar defects were observed in Fmn2b morphants injected with MO TB Fmn2b (17.7 ms; [16.50,19.00]; *n* = 82; Extended Data Fig. 2-1*D*).

Previous reports implicate M-cells in mediating short latency (SLC; <13 ms) escapes in response to mechano-acoustic stimuli (Lacoste et al., 2015). Whereas, long latency escapes are generally non-Mauthner-mediated responses. We classified the latency to the first movement as short latency C-bend (SLC; <13 ms) and long latency C-bend (LLC; 13–26 ms) events. Of the total number of events, we observed a decrease in the fraction of SLC versus LLC responses in Fmn2b morphants. In morphants co-injected with mFmn2-GFP mRNA, the fraction of events showing SLC responses is greater than LLC responses as seen in control morphants (Fig. 2*C*,*D*).

However, the latency to achieve maximum C-bend escape angle (Fig. 2*E*) and the maximum escape angle (Fig. 2*F*) were comparable between the control morphants, the Fmn2b morphants and the Fmn2b morphants co-injected with mouse *fmn2* mRNA.

Hence, Fmn2b knock-down increased the latency to respond to mechano-acoustic stimuli although the responsiveness and the ability to elicit an escape response remains uncompromised. These results suggest a specific and perhaps localized defect in the mechano-acoustic response circuitry.

### Fmn2b depletion does not affect the sensory components of the acoustic startle circuit

**Figure 3.**
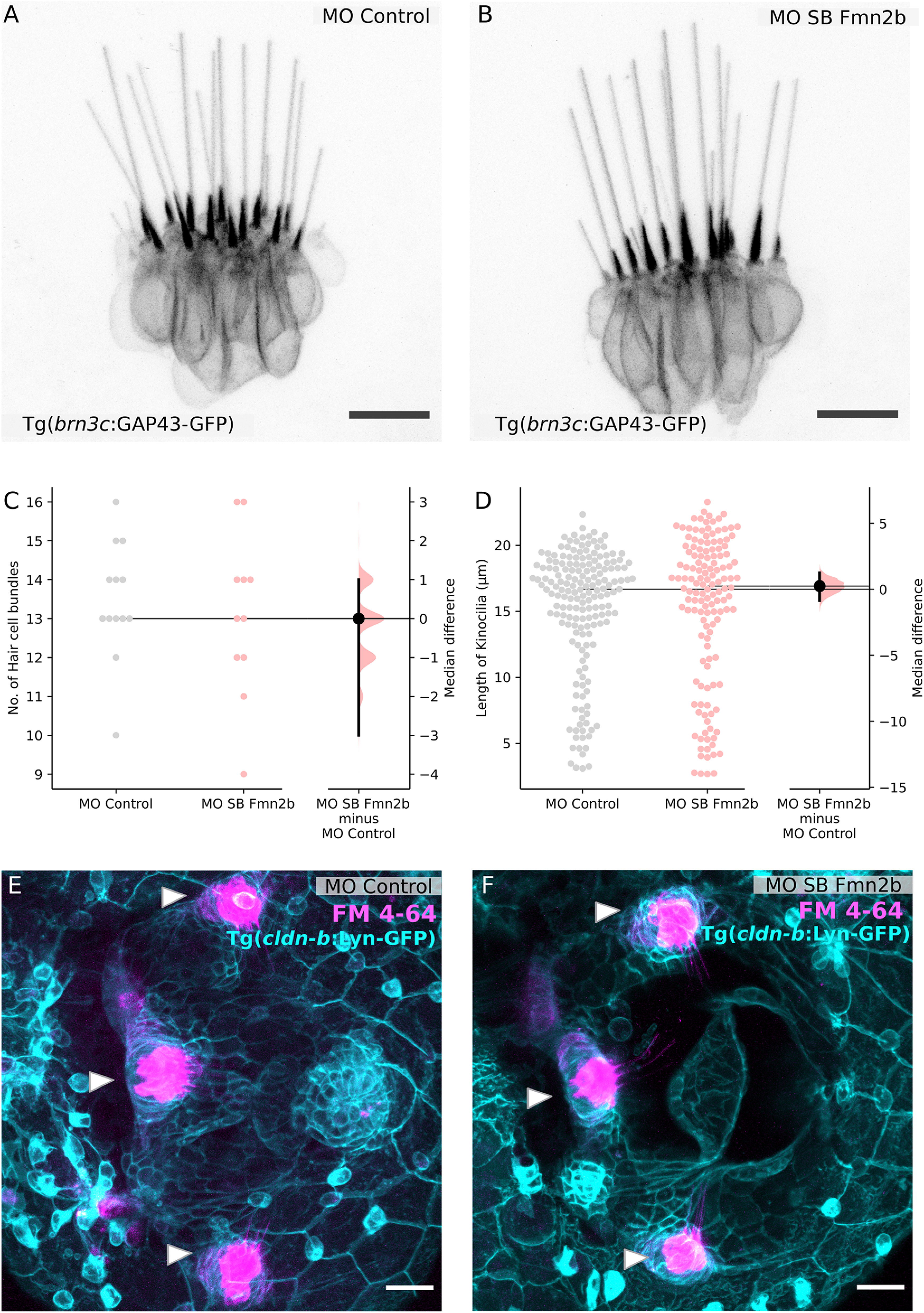
Sensory components of the acoustic startle circuit are not affected in Fmn2b morphants. Hair cells of the lateral crista of the zebrafish inner ear are visualized using the Tg (*brn3c*:GAP43-GFP) line in 96-hpf larvae with (***A***) 2 ng MO control and (***B***) 2 ng MO SB Fmn2b cytoplasmic injections. Scale bar: 10 μm. ***C***, Quantification of the number of hair cell bundles with a kinocilium is depicted in the Gardner–Altman plot. The unpaired median difference between MO control and MO SB Fmn2b is 0.0 [95.0%CI −3.0, 1.0]. The *p* value of the two-sided permutation *t* test is 0.687. ***D***, Quantification of kinocilia length in the lateral crista hair cells is shown in the Gardner–Altman plot. The unpaired median difference between MO control and MO SB Fmn2b is 0.247 [95.0%CI −0.851, 1.25]. The *p* value of the two-sided permutation *t* test is 0.663. For all the estimation statistics analysis, the effect sizes and CIs are reported as effect size [CI width lower bound; upper bound]. Representative images for FM-4-64 dye uptake assay in the inner ear of 96-hpf (***E***) control morphants and (***F***) Fmn2b morphants, done in the background of Tg(*cldnb*:lyn-GFP) to mark the inner ear boundary. Scale bar: 20 μm.

The acoustic startle response is essential for the survival of zebrafish larvae in terms of reacting to the environment efficiently and reliably. The behavioral deficits observed in Fmn2b morphants could arise from one or more components of the acoustic startle circuit. We systematically evaluated the different components of the acoustic startle circuit to identify the origins of the delay in the initiation of the C-bend escape in Fmn2b morphants. M-cells receive auditory input from the inner ear hair cells, relayed by the statoacoustic ganglion (SAG; Whitfield et al., 2002; Medan and Preuss, 2014). Evaluation of the otic vesicle and the otoliths did not reveal any anatomic or structural defects. We probed the structural integrity of the inner ear hair cells responsible for the mechanotransduction of the acoustic cues, using the Tg(*brn3c*:GAP43-GFP) line. This line labels a subset of the hair cells in the inner ear and lateral line neuromasts (Xiao et al., 2005). Microscopic analysis of kinocilia and hair cell bundles of the inner ear cristae revealed no significant differences between control and Fmn2b morphants (Fig. 3*A–D*). Uptake of the lipophilic dye FM-4-64 was used to evaluate the functional activity of the inner ear hair cells (Pacentine and Nicolson, 2019). These experiments indicated that activity-dependent vesicle recycling was comparable between control and Fmn2b morphants (Fig. 3*E*,*F*) and suggested that the synaptic activity at the hair cell ribbon synapses is mostly unaffected in the Fmn2b morphants.

**Figure 4.**
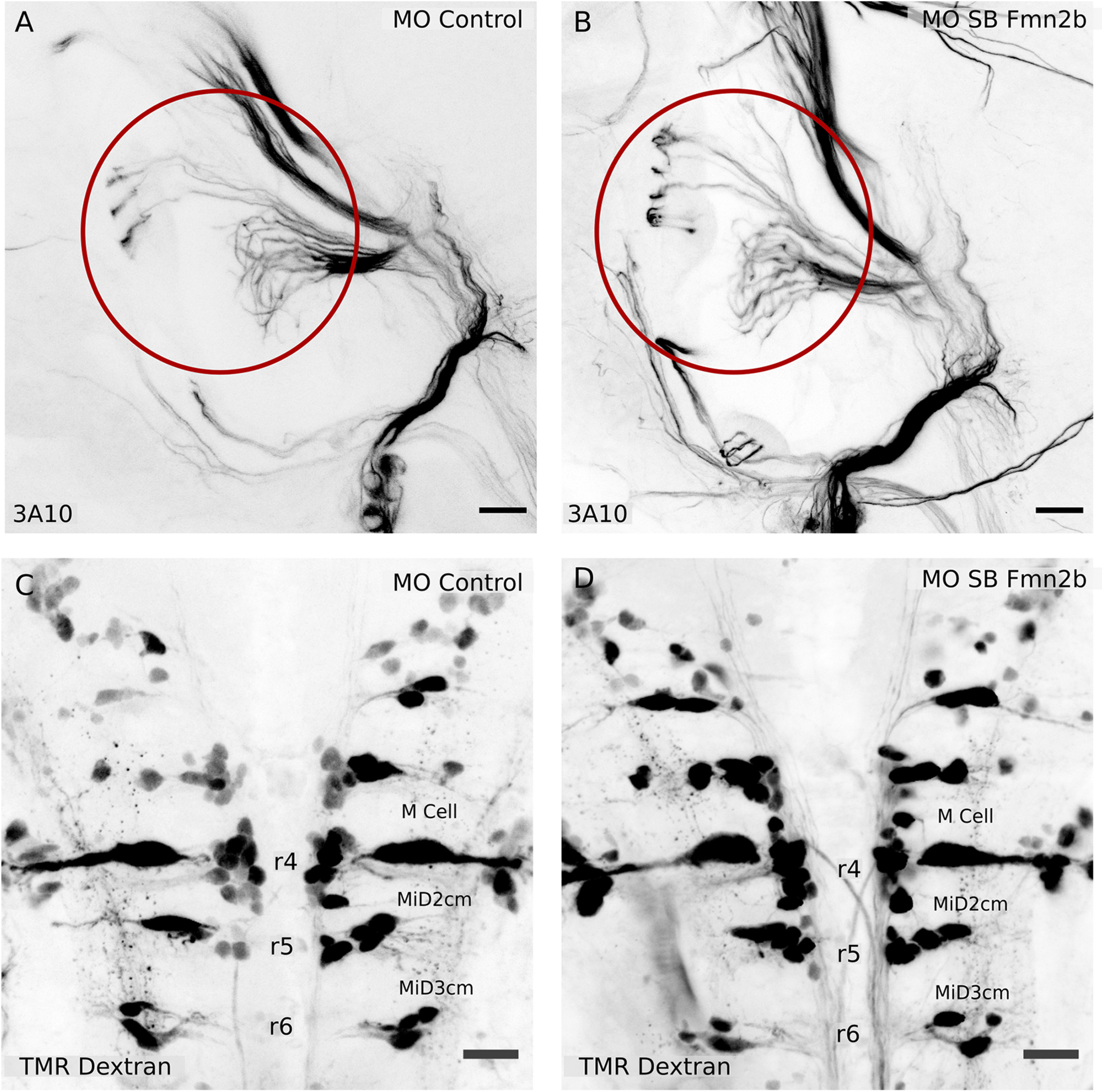
The SAG relaying sensory information and the hindbrain reticulospinal neurons are not affected in Fmn2b morphants. Whole-mount immunostaining with anti-neurofilament antibody 3A10 was used to visualize the SAG connecting to the M-cells in 96-hpf larvae with (***A***) 2 ng MO control and (***B***) 2 ng MO SB Fmn2b cytoplasmic injections. Scale bar: 20 μm. Retrograde labeling of reticulospinal neurons using TMR dextran in 96-hpf (***C***) 2 ng MO control-injected embryos and (***D***) 2 ng MO SB Fmn2b-injected embryos. The M-cell and its homologs, MiD2cm and MiD3cm, are intact in Fmn2b morphants. Scale bar: 20 μm. The cell bodies of the reticulospinal neurons stained with RMO-44 antibody in 48-hpf morphants are intact in the control and Fmn2b morphants (Extended Data Fig. 4-1).

The next component of the acoustic startle circuit is the SAG, which connects the inner ear hair cells to the M-cell. SAG was also found to be structurally unperturbed as evident in 96-hpf morphants immunostained with anti-neurofilament 3A10 antibody (Fig. 4*A*,*B*). Further, we labeled the reticulospinal neurons in the hindbrain of 4-dpf morphants to assess the integrity of the M-cell and its homologs MiD2cm and MiD3cm, using TMR dextran. The M-cell and its homologs, primarily responsible for regulating acoustic startle responses were found to be intact in Fmn2b morphants (Fig. 4*C*,*D*). We also performed immunostaining with 48-hpf morphants using another anti-neurofilament antibody RMO-44, which has better reactivity than 3A10 for hindbrain cell bodies. We found that the cell bodies and axonal tracts were comparable between the control and Fmn2b morphants (Extended Data Fig. 4-1*A*,*B*).

10.1523/ENEURO.0329-20.2021.f4-1 Extended Data Figure 4-1 Reticulospinal neuron cell bodies remain unaffected in Fmn2b morphants. Whole-mount immunostaining using RMO-44 antibody of 48-hpf (***A***) 2 ng MO control morpholino injected and (***B***) 2 ng MO SB Fmn2b morpholino-injected embryos shows that there are no significant changes in the cell bodies of reticulospinal neurons at early stages. Scale bar: 20 μm. Download Figure 4-1, TIF file.

Our findings suggest that the sensory components of the acoustic startle circuit providing input to the M-cell are unaffected by Fmn2b knock-down. This observation is consistent with the unaffected responsiveness to mechano-acoustic stimuli in Fmn2b morphants. These results suggest that the behavioral defect of increased latency is caused by deficits in the neural circuit downstream of the SAG. We found the reticulospinal neuron cell bodies including the M-cell and its homologs to be intact in Fmn2b morphants. This encouraged us to probe the inputs to the M-cell in the zebrafish hindbrain in detail.

### The development of spiral fiber tracts in the hindbrain is regulated by Fmn2b

**Figure 5.**
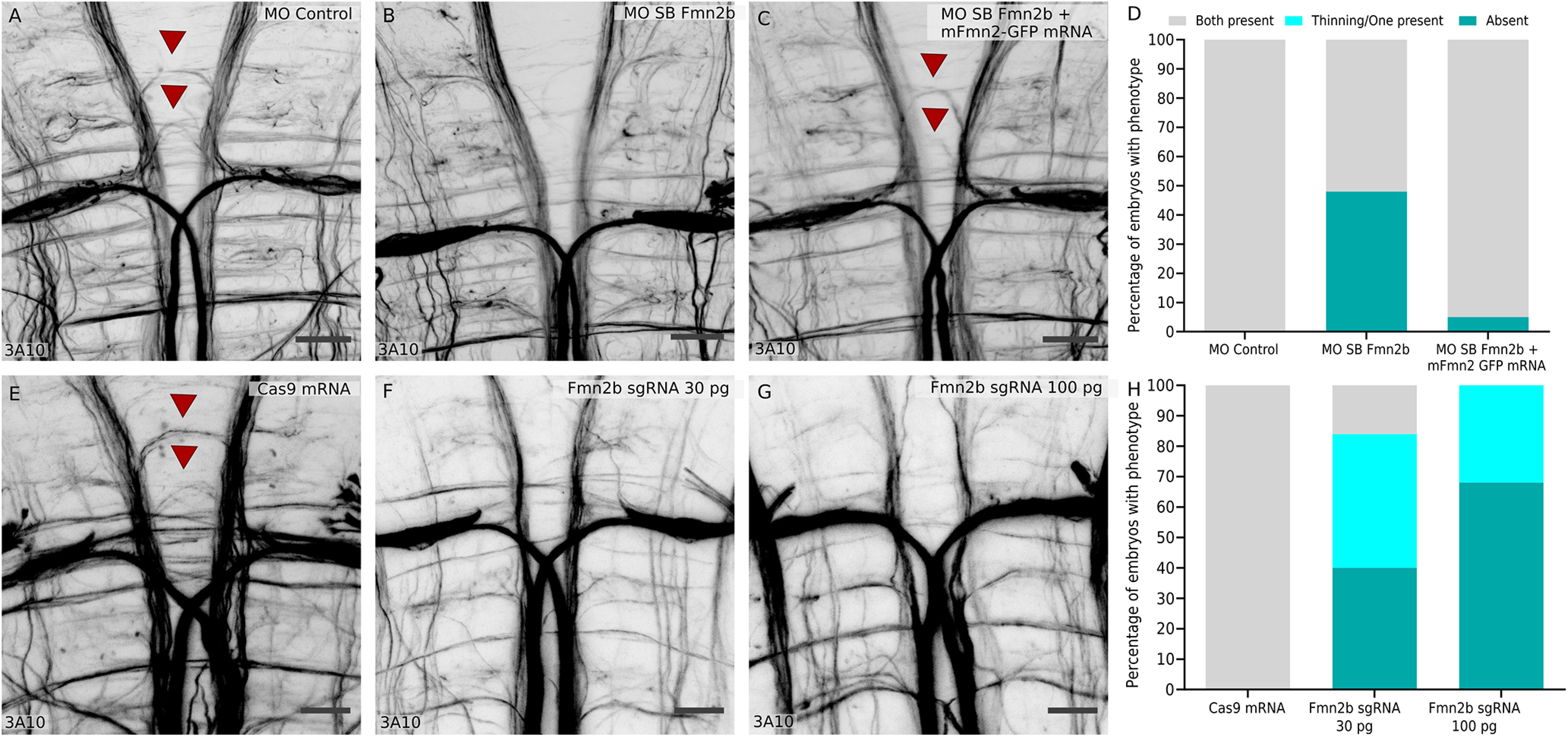
Fmn2b knock-down impairs axonal outgrowth in the spiral fiber tracts. Whole-mount immunostaining using anti-neurofilament antibody 3A10 stains axons in 96-hpf zebrafish hindbrain (***A–C***, ***E–G***). ***A***, Spiral fiber tracts are marked with red arrowheads in control morphants. ***B***, Fmn2b knock-down using splice blocking morpholino (MO SB Fmn2b; 2 ng/embryo injected in the cytoplasm) reveals defects in the spiral fiber tract outgrowth. The spiral fiber defect is recapitulated with the translation blocking morpholino MO TB Fmn2b in a dose-dependent manner (Extended Data Fig. 5-1*A*,*B*). ***C***, The phenotype in Fmn2b morphants could be rescued by injection of 300 pg mFmn2-GFP mRNA in the MO SB Fmn2b-injected embryos at one cell stage. Scale bar: 20 μm. ***D***, A total of 48% Fmn2b morphants (*n* = 31) show the absence of spiral fiber tracts as compared with none in the control morphants (*n* = 27). However, only 5% of the embryos rescued with mFmn2-GFP mRNA (*n* = 40) showed the defects. A χ^2^ test of independence was performed to compare the three groups, χ^2^ (df = 2) = 95.75, *p* < 0.0001. ***E***, Spiral fiber neurons marked with red arrowheads in embryo injected with Cas9 mRNA. ***F***, Fmn2b knock-down using 30-pg dose of each of the two sgRNAs against *fmn2b* causes defects in the outgrowth of the spiral fiber tracts in Fmn2b crispants. ***G***, Fmn2b knock-down using 100-pg dose of each of the two sgRNAs against *fmn2b* causes defects in the outgrowth of the spiral fiber tracts in Fmn2b crispants. Scale bar: 20 μm. ***H***, Quantification of the phenotype reveals that Fmn2b crispants injected with 30-pg sgRNAs and Cas9 mRNA (*n* = 25) show absence of both the spiral fiber tracts in 40% embryos and thinning or absence of only one tract in 44% embryos, as compared with no defects observed in the group injected with the Cas9 mRNA only (*n* = 27). Fmn2b crispants injected with 100-pg sgRNAs and Cas9 mRNA (*n* = 28) show an increase in the severity of the phenotype with 67.85% embryos showing absence of both tracts, 32.15% showing thinning or single tract and no embryos with both the spiral fiber tracts intact. A χ^2^ test of independence was performed to compare the three groups, χ^2^ (df = 4) = 255, *p* < 0.0001. Representative images of the single spiral fiber tract outgrowth and thinning of the spiral fiber tract in Fmn2b crispants are included in the Extended Data in comparison to Cas9 mRNA-injected embryos (Extended Data Fig. 5-1*C–E*). Crispants showed similar morphologic defects during early development when compared with morphants (Extended Data Fig. 5-1*F*). Various indels and base changes introduced in crispants by co-injection of sgRNA-1 and sgRNA-2 along with Cas9 mRNA are summarized in Extended Data Figure 5-2*A*,*B*.

The M-cell is known to receive inputs from excitatory as well as inhibitory interneurons (Korn and Faber, 2005) to achieve modular fine-tuning of responses to a variety of stimuli. The increase in the response latency in Fmn2b morphants suggests possible defects in the hindbrain circuits involving the M-cells. To assess the state of neuronal connectivity in the hindbrain, we visualized the axonal tracts in 96-hpf morphants using the anti-neurofilament antibody 3A10. We found that the majority of the axonal tracts in the hindbrain, including the M-cell and its homologs, remain unaffected in Fmn2b morphants. However, the spiral fiber tracts in rhombomere three were absent. Spiral fiber neurons are commissural interneurons innervating the M-cell axon hillock (Scott et al., 1994; Lorent et al., 2001; Gyda et al., 2012; Lacoste et al., 2015; Liu and Hale, 2017). They have been described earlier to provide excitatory feedforward input to the M-cells and regulate the escape response in larval zebrafish (Lacoste et al., 2015). We found that 48% of the MO SB Fmn2b morphants (*n* = 31) showed absence of spiral fiber tracts, whereas none of the control morphants (*n* = 27) showed the defect (Fig. 5*D*). The phenotype persisted in 36% of the embryos injected with a 2-ng dose of the second morpholino, MO TB Fmn2b at one-cell stage (Extended Data Fig. 5-1*A*,*B*). Further, yolk injections of higher doses (4 and 8 ng) of the MO TB Fmn2b caused similar spiral fiber tract development defects. We were also able to rescue the phenotype by co-injecting mFmn2-GFP mRNA along with MO SB Fmn2b. The phenotype was rescued in 95% (*n* = 40) of the morpholino and mFmn2-GFP mRNA-co-injected embryos.

10.1523/ENEURO.0329-20.2021.f5-1 Extended Data Figure 5-1 The translation blocking morpholino recapitulates the spiral fiber neuron defect in Fmn2b morphants. ***A***, Whole-mount immunostaining using 3A10 antibody of 96-hpf Fmn2b morphants injected with 2 ng MO TB Fmn2b phenocopies the splice blocking Fmn2b morphant defects. Scale bar: 20 μm. ***B***, Quantification of cytoplasmic injections of 2 ng MO TB Fmn2b and yolk injections of higher doses (4 and 8 ng) of MO TB Fmn2b. Both cause the spiral fiber neuron outgrowth defect in a dose-dependent manner. Representative micrographs showing the (***D***) absence of only one tract and (***E***) thinning of the spiral fiber tract in Fmn2b crispants as compared to (***C***) embryos injected with only Cas9 mRNA. ***F***, Quantification of morphological defects in Fmn2b crispants injected with 100 pg each of sgRNA1 and sgRNA2 along with 300 pg of Cas9 mRNA at 96 hpf. The parameters used for classification of morphologically aberrant embryos were the same as in Extended Data Figure 2-1*F*. Download Figure 5-1, TIF file.

10.1523/ENEURO.0329-20.2021.f5-2 Extended Data Figure 5-2 Summary of indels and base changes generated in Fmn2b crispants. Out of 15 randomly chosen crispants injected with sgRNA-1, sgRNA-2 and Cas9 mRNA, (***A***) 93.3% embryos exhibited indels at the sgRNA-1 locus, and (***B***) 53.3% embryos showed indels at the sgRNA-2 locus on exon 1 of *fmn2b*. Remaining larvae also exhibited base changes at the both the loci. in: insertion; del: deletion. Download Figure 5-2, TIF file.

Further, we injected embryos with two sgRNAs targeting the exon1 of *fmn2b* along with Cas9 mRNA to investigate whether knock-down of Fmn2b using CRISPR-Cas9 based mosaic indels would phenocopy the defects in spiral fiber tract development observed on morpholino-based knock-down. 3A10 immunostaining revealed dose-dependent spiral fiber tract defects in the crispants. In the group which received a 30-pg dose of sgRNAs (*n* = 25), 40% of the embryos had no spiral fiber tracts while another 44% had either only one spiral fiber tract or significant thinning of the tracts. In the group injected with the 100-pg sgRNA dose (*n* = 28), 67.85% of the embryos showed absence of both the spiral fiber tracts while another 32.15% either lacked one tract or had thin tracts. At this dose, no larvae had intact spiral fiber tracts. The dose-dependent prevalence of the spiral fiber tract development defect reinforces the requirement of Fmn2b in the development of spiral fiber neuron tracts. As seen in morphants, 30–40% of the Fmn2b crispants also showed morphologic defects like, curved body axis, shorter body lengths, cardiac edema, microcephaly, deflated swim bladder and otolith defects. The morphologic defects observed in morphants and crispants have been quantified in Extended Data Figures 2-1*E* and 5–1*F*, respectively.

These results implicate Fmn2b in the development of the spiral fiber neuron tracts that are necessary for the efficient and reliable execution of the M-cell-mediated C-bend escape response. The increase in latency to respond to mechano-acoustic stimulus in Fmn2b morphants can be attributed to the lack of innervation of the M-cells by excitatory spiral fiber neurons.

## Discussion

The zebrafish *fmn2* ortholog, *fmn2b*, was identified to be located on chromosome 12. The three key functional protein domains at the C terminus (FH1, FH2, and FSI domains) that define the fly, chick, mouse, and humans orthologs of Fmn2 (Higgs, 2005; Breitsprecher and Goode, 2013) were found to be highly conserved in zebrafish. On the other hand, the N-terminal region was less conserved across these species, perhaps reflecting species-specific regulatory diversity. However, the ability of mouse *fmn2* mRNA to rescue the Fmn2b depletion induced phenotypes in zebrafish underscores the functional conservation between these species.*fmn2b* mRNA is enriched in the developing zebrafish nervous system though the expression pattern is dynamic during early development. *fmn2b* mRNA is maternally deposited in embryos but disappears after 3 hpf. Robust expression of *fmn2b* mRNA in the nervous system resumes by 24 hpf and persists until 96 hpf (Fig. 1*B–J’*). The spatiotemporal expression pattern coincides with neuronal development in the zebrafish embryo.

Fmn2b morphants and crispants exhibit gross morphologic defects like body curvature, short body length, microcephaly, cardiac edema, swim bladder, and otolith defects. Oocytes from Fmn2 null mice are known to have spindle positioning defects and meiotic arrest causing hypo-fertility in Fmn2 mutants (Leader and Leder, 2000; Mogessie and Schuh, 2017). Early developmental defects in Fmn2b morphants and crispants could be because of similar defects. The consistent appearance of morphologic defects, mostly related to early development and predominantly in non-neuronal tissues, in morphants as well as crispants suggests that Fmn2b has pleiotropic functions during the development of zebrafish embryos. However, ∼60% of the embryos show normal development. We used morphologically normal embryos for all the experiments in this study to avoid behavioral assays being influenced by morphologic deficits.

Behavioral analysis using an automated stimulus delivery setup and high-speed recording revealed a specific function of Fmn2b in the acoustic startle response. The responsiveness of Fmn2b and control morphants were comparable and indicated that sensory perception was unaffected. However, Fmn2b knock-down increased the latency to respond; the proportion of fast responses decreased while the long latency escape responses increased (Fig. 2*B–D*).

Consistent with the behavioral observations, the inner ear hair cells (Fig. 3*A–F*) and the SAG (Fig. 4*A*,*B*) were found to be unaffected by Fmn2b depletion. Strikingly, we found that spiral fiber neurons, which provide excitatory input to the M-cells and form synaptic terminals at the M-cell axon hillock fail to extend their tracts across the midline (Fig. 5*A*,*B*). Thus, the observed deficits in behavior are likely because of the absence of spiral fiber neuron innervation resulting in the failure of the command neuron-like M-cells to reach the excitatory threshold (Lacoste et al., 2015). We were able to rescue both the behavioral (Fig. 2*B–D*) and the neuro-anatomic phenotypes in Fmn2b morphants by co-injection of mFmn2-GFP mRNA (Fig. 5*C*). This result underscores the evolutionarily conserved function of Fmn2 from teleosts to mammals.

Previous studies have highlighted the role of spiral fiber neurons in regulating the fast escape responses in response to mechano-acoustic stimuli (Lorent et al., 2001; Lacoste et al., 2015; Hale et al., 2016; Marsden et al., 2018). Spiral fiber neurons directly respond to mechano-acoustic stimuli and relay the information to the M-cells, which also receive direct sensory inputs (Lacoste et al., 2015). In a separate study, the absence of spiral fiber neurons along with other hindbrain commissures caused locomotor defects in *space cadet* mutants (Lorent et al., 2001) later characterized as a mutation in the retinoblastoma-1 (Rb1) gene (Gyda et al., 2012). These studies indicate that the spiral fiber neurons are indispensable for the initiation of a robust startle escape response (Hale et al., 2016). The convergent circuit design in zebrafish hindbrain, where spiral fiber neurons help M-cells reach the excitatory threshold, ensures a speedy and reliable response to a potentially noxious stimulus. The excitability of M-cells decides the further course of action for the animal within the specific context.

Zebrafish need the M-cells and two segmental homologs, MiD2cm and MiD3cm, to elicit an effective, fast escape response in zebrafish (Liu and Fetcho, 1999; Kohashi and Oda, 2008). These three neurons receive a common auditory input but can generate different outputs, evident by their different spiking properties, to downstream neurons to control adaptive escape behaviors. MiD2cm and MiD3cm mediate escapes with longer latencies as compared with the M-cell (Nakayama and Oda, 2004; Kohashi et al., 2012; Lacoste et al., 2015). The M-cell homologs ensure that an escape response is elicited in the absence of M-cell firing or for weaker stimuli. Ablation of the M-cell, especially the axon initial segment, causes an increase in latency of response to mechano-acoustic stimuli (Liu and Fetcho, 1999; Hecker et al., 2020).

In Fmn2b morphants, the M-cells fail to receive inputs from the spiral fiber neurons and exhibit a delay but eventually elicit an escape response. It is likely that MiD2cm and MiD3cm neurons, which also receive the auditory input from the SAG, are recruited in Fmn2b morphants to produce an escape response with increased latency. In addition to the overall increase in latency in Fmn2b morphants, the shift toward majority LLC responses in Fmn2b morphants instead of SLC responses implies the involvement of M-cell homologs in the absence of spiral fiber excitation of the M-cell.

While the possibility of further deficits downstream of the M-cell homologs cannot be ruled out, the Fmn2b morphants can still execute C-bend escapes with no significant changes in the maximum bending angle in response to mechano-acoustic stimuli. Therefore, we conclude that the absence of M-cell innervation by the spiral fiber neurons are the primary reason for the behavioral deficits in Fmn2b morphants.

Regulated and adaptive remodeling of the neuronal cytoskeleton is central to almost all aspects of neural development, including neurogenesis, neurite initiation, growth cone-mediated pathfinding and synaptogenesis (Lowery and Van Vactor, 2009; Flynn, 2013; Gordon-Weeks and Fournier, 2014; Muñoz-Lasso et al., 2020). The dynamics of the neuronal cytoskeleton is mediated by a complex interplay between the different cytoskeleton components, each regulated by specific regulators but also coordinated by co-regulatory activities. The dynamics actin cytoskeleton is regulated by several actin-binding proteins, and mutations in many of these have been associated with neurodevelopmental disorders (Lian and Sheen, 2015; Muñoz-Lasso et al., 2020).

Actin nucleators are an important class of actin-binding proteins that are involved in regulating the seeding of F-actin filaments from monomeric actin and control the architecture of the F-actin network. Three classes of actin nucleators have been described previously in zebrafish, Arp2/3 complex, Formin homology domain 2 (FH2) containing family (the Formins) and the WASP homology domain 2 (WH2) containing family of nucleators. Arp2/3 has been shown to regulate actin patches essential for proximal axon specification in zebrafish motor neurons (Balasanyan et al., 2017) and maintenance of microridge structure and length on surface epithelial cells in zebrafish (Lam et al., 2015; Pinto et al., 2019). The WH2 domain containing actin nucleator, Cordon bleu (Cobl), is required for the development of motile cilia in zebrafish Kuppfer’s vesicles which in turn maintain laterality in zebrafish (Ravanelli and Klingensmith, 2011).

There are fifteen formins in humans that cluster into eight different subfamilies (Schönichen and Geyer, 2010) and mutations in several formins are associated with a variety of neural disorders (Kawabata Galbraith and Kengaku, 2019). Most formins are conserved across vertebrates and are expressed in a variety of tissues, including the nervous system (Dutta and Maiti, 2015). The formin family comprises of 25 predicted members in zebrafish out of which 11 are shown to be neuronally enriched (Thisse and Thisse, 2005; Santos-Ledo et al., 2013). A study comparing expression patterns of the formin-like (fmnl) subfamily of formins shows that the formins, fmnl1a, fmnl1b, fmnl2a, fmnl2b, and fmnl3 are expressed in the nervous system in a dynamic temporal manner during development, in addition to expression in non-neuronal tissues (Santos-Ledo et al., 2013). However, the role of formins in the development of neural circuits in zebrafish is not well explored despite several studies reporting the enrichment of formins in vertebrate nervous systems (Leader and Leder, 2000; Krainer et al., 2013; Dutta and Maiti, 2015; Sahasrabudhe et al., 2016). The only report available implicates the formin Daam1a in the asymmetric morphogenesis of the zebrafish habenula and the regulation of the dendritic and axonal processes of the dorsal habenular neurons (Colombo et al., 2013). Formin function in non-neuronal tissues has also been sparsely investigated in zebrafish. Daam1a is required for convergent extension and regulates notochord development in zebrafish (Kida et al., 2007) while zDia2, working synergistically with Profilin I, regulates gastrulation (Lai et al., 2008). Fmnl3 has been reported to be involved in blood vessel morphogenesis (Phng et al., 2015).

Formins are expressed in several tissue types with rich spatiotemporal diversity in humans (Krainer et al., 2013), mice (Dutta and Maiti, 2015), and zebrafish (Thisse and Thisse, 2005; Kida et al., 2007; Lai et al., 2008; Santos-Ledo et al., 2013; Sun et al., 2014). The conservation of multiple of formins with overlapping expression in the nervous system possibly reflects a diversity of distinct regulatory functions, ensuring the highly adaptive yet specific cytoskeleton remodeling necessary for accurate circuit development.

Given the broad expression of the *fmn2b* mRNA in the developing and adult zebrafish nervous system, it is interesting to note that the defects because of Fmn2b knock-down are confined to the development of a small population of hindbrain excitatory interneurons, the spiral fiber neurons.

Spiral fiber neurons are late pioneering neurons which complete axonogenesis around 72 hpf (Lorent et al., 2001). The developmental timing of axonal outgrowth in this neuronal population and protein perdurance from the maternal *fmn2b* mRNA may render spiral fiber neurons, especially sensitive to morpholino-mediated knock-down. There is a notable expression of *fmn2b* mRNA in the RGCs of the eye and the spinal cord of zebrafish larvae. However, the role of Fmn2b in development of other neural circuits in zebrafish remains untested.

Fmn2 has previously been shown to be necessary for the axonal outgrowth of spinal neurons in developing chick embryos (Sahasrabudhe et al., 2016; Ghate et al., 2020). Similarly, we speculate that the zebrafish spiral fiber neurons have axonal outgrowth defects that result in the loss of synaptic connectivity with the M-cells. However, it remains possible that there are additional deficits in neuronal differentiation or specification in the Fmn2b morphants.

Both Fmn2b morphants and crispants show defects in the development of the spiral fiber tract. Given the mosaic nature of F0 CRISPR-Cas9-mediated knock-out, the thinning of the axonal tract on Fmn2b knock-down supports our speculation of axonal outgrowth defect in spiral fiber neurons. The dose-dependent effect of the sgRNAs on spiral fiber tract development further strengthens our claim that the spiral fiber neurons have axonal outgrowth defects. However, it remains to be tested whether in addition to outgrowth defects, Fmn2b morphants have neural differentiation or specification defects. Previous studies show that Fmn2 has a synergistic effect on Filamin-a (Flna) and causes neurodevelopmental defects in Fmn2 and Flna double knock-out mice. On its own, Fmn2 null allele does not cause neurodevelopmental defects, neural differentiation defects or apoptotic phenotypes in Fmn2 single knock-out mice (Lian et al., 2016). In zebrafish Fmn2b morphants, we show that the cell bodies of the reticulospinal neurons in the hindbrain labeled by TMR dextran retrograde labeling (Fig. 4) and RMO-44 immunostaining (Extended Data Fig. 4-1) are intact in the Fmn2b morphants. This indicates that Fmn2, consistent with the findings in primary neuron cultures, is more likely to be involved in the axon outgrowth of spiral fiber neuron tracts and not in the specification or differentiation of the neurons.

The molecular mechanism mediating Fmn2b-dependent axonal outgrowth in zebrafish is not known. In chick spinal neurons, Fmn2 mediates growth cone motility by regulating the cell-matrix adhesions necessary to generate traction forces (Sahasrabudhe et al., 2016; Ghate et al., 2020). A recent study implicates Fmn2b in regulating growth cone microtubule dynamics in zebrafish Rohon–Beard neurons (Kundu et al., 2021) and highlights another mechanism mediating outgrowth and pathfinding. Further studies in zebrafish, involving *in vivo* imaging of cytoskeletal dynamics, can pave the way for mechanistic insights into Fmn2 function in intact animals.

In recent years, mutations in Fmn2 have been increasingly associated with neural disorders including cognitive disabilities and sensory processing dysfunction in humans (Perrone et al., 2012; Almuqbil et al., 2013; Law et al., 2014; Agís‐Balboa et al., 2017; Anazi et al., 2017; Marco et al., 2018; Gorukmez et al., 2020). Fmn2 expression was found to be reduced in postmortem brain samples of patients with posttraumatic stress disorder and Alzheimer’s disease (Agís‐Balboa et al., 2017). Other reports implicate Fmn2 mutations in corpus callosum agenesis (Perrone et al., 2012; Gorukmez et al., 2020) and microcephaly (Anazi et al., 2017). In rodents, loss of Fmn2 accelerated age-associated memory impairment and amyloid-induced deregulation of gene expression (Peleg et al., 2010; Agís‐Balboa et al., 2017). Despite accumulating evidence, little is known about the function of Fmn2 in the nervous system. A zebrafish Fmn2b loss-of-function model can provide valuable neurodevelopmental insights into Fmn2 function.

This study explores Fmn2 function in a vertebrate model and links the axonal development of an identified group of neurons to a specific behavioral deficit. Fmn2b was found to mediate the development of the spiral fiber neuron pathway conveying indirect excitatory inputs to the command neuron-like M-cells. The loss of this specific regulatory unit is manifested in a delay in initiating fast escape reflexes in response to mechano-acoustic stimuli, a behavior of significant survival value. Apart from identifying a novel function for Fmn2 in the development of hindbrain commissural circuitry, our findings highlight the utility of models bridging subcellular functions of Fmn2 identified in cultured neurons to circuit development and associated behavioral consequences.

## Synthesis

Reviewing Editor: Lynda Erskine, University of Aberdeen Decisions are customarily a result of the Reviewing Editor and the peer reviewers coming together and discussing their recommendations until a consensus is reached. When revisions are invited, a fact-based synthesis statement explaining their decision and outlining what is needed to prepare a revision will be listed below. The following reviewer(s) agreed to reveal their identity: Thomas Becker.

Thank you for submitting the revised version of your manuscript. The reviewers both acknowledged the considerable effort that went into preparing this revised version. However, while the additional work and changes to the manuscript have addressed many of the points in the original reviews, this has also raised additional issues that require to be resolved. Below are the points that, following discussion between the reviewers and myself, we are all agreed need addressing.

Major points:

1. A major concern is that knocking down a gene by injection to varying degrees with both MO and gRNA and then picking the larvae that are only mildly affected (40% for each treatment are apparently malformed) is not the best approach. With gRNAs, toxicity is usually low, so this points to a necessary early function of this cytoskeletal gene, such that a specific function in the spiral fibre context cannot be separated out.

A full discussion of the early phenotypes in the fmn2 deficient animals and how that potentially impacts on their later phenotype analysis is required, such that the reader can fully evaluate the outcome of the experiment.

2. There is no control for the efficacy of the gRNAs. How do the researchers know that the gene is rendered non-functional by the injections? Did the authors sequence the mutations generated by the gRNAs in exon 1?

3. The morphological phenotypes for both morphants and crispants only received a score as either having a phenotype or not. Scoring is not a robust way of assessing phenotypes that needs strong controls. In addition, there is no mention of making the scorer unaware of the expected outcome (“blinding”) of the assessment. In combination, scoring and lack of blinding make phenotype quantification open to a very high level of experimental bias.

The authors should provide more quantitative analysis of the phenotypes. For example, they could first binarize their images in ImageJ (using a consistent threshold) before analysing the presence or absence of spiral tract or quantifying the signal in another way in a blind manner.

4. Phenotypes are described in the text that are not depicted in the images. For example, a “significant thinning of the tracts” is described, but this is not convincingly shown or quantified in Fig. 5. Moreover, it is stated that in line 452f of the discussion that spiral fibres fail to form synaptic terminals at the M-cell axon hillock. This is not described in the results and not visible in the images.

Minor points:

1. As the authors mentioned in their reply, Fmn2 should be written with a first letter upper case and not italicized when describing the protein. Many sentences in the introduction describe the protein and its functions. They should therefore use Fmn2 rather than fmn2, as it was written in the first submission (lines 5, 27, 70-79, etc).

2. Typos / wording:

- Line 39: remove “the” between “during” and “development"

- Line 92: remove “the” before “fmn2 expression"

- Line 157: add “of” between “pg” and “both"

- Lines 385: remove “the axonal tracts of” (redundant with “the Spiral fiber tracts”), or replace “the Spiral fiber tracts” by “the Spiral fiber neurons"

## Author Response

Synthesis Statement for Author (Required):

Thank you for submitting the revised version of your manuscript. The reviewers both acknowledged the considerable effort that went into preparing this revised version. However, while the additional work and changes to the manuscript have addressed many of the points in the original reviews, this has also raised additional issues that require to be resolved. Below are the points that, following discussion between the reviewers and myself, we are all agreed need addressing.

We thank the editor and the reviewers for appreciating our efforts. The criticism received have improved the manuscript significantly. We have addressed all the concerns raised and hope these will be acceptable.

Major points:

1. A major concern is that knocking down a gene by injection to varying degrees with both MO and gRNA and then picking the larvae that are only mildly affected (40% for each treatment are apparently malformed) is not the best approach. With gRNAs, toxicity is usually low, so this points to a necessary early function of this cytoskeletal gene, such that a specific function in the spiral fibre context cannot be separated out.

A full discussion of the early phenotypes in the fmn2 deficient animals and how that potentially impacts on their later phenotype analysis is required, such that the reader can fully evaluate the outcome of the experiment.

As indicated by the reviewer the toxicity of gRNAs is typically is low. As we see a similar extent of morphological defects with gRNA and morpholinos, these defects are likely to be attributable to loss of Fmn2b function.

To give the reader a clearer picture, we are now including the quantification of the morphological defects seen in Fmn2b morphants and crispants (Extended data Fig 5-1 E, F). We find that the morphological defects persist in similar proportions in both the morphant and crispant populations. The reason for choosing morphologically intact larvae is to avoid biases driven by these morphological deficits in the locomotion-based assays. As the spiral fiber neurons begin axonogenesis relatively late in development (around 72 hpf) (Lorent et al., 2001), the influence of the general and early developmental deficits is expected to be minimal (at least in morphologically intact larvae). It is certainly possible that there may be defects in the development of other neural circuits that have not been identified in our study. However, the specific change in the short latency response in the startle reflex is closely correlated with the loss of spiral fiber activity (Lacoste et al., 2014).

We have added a section discussing the defects during early development in the revised version of the manuscript.

2. There is no control for the efficacy of the gRNAs. How do the researchers know that the gene is rendered non-functional by the injections? Did the authors sequence the mutations generated by the gRNAs in exon 1?

We co-injected two sgRNAs and Cas9 mRNA to target exon 1 of fmn2b. The genomic DNA from 24 hpf crispants was used for PCR amplification of the region flanking the two sgRNAs and Sanger sequencing was performed on the amplicons. For the sgRNA-1 locus, 93.3% crispants showed indels and for the sgRNA-2 locus, 53.3% crispants showed indels. The indels are summarized in Figure 5-2. Moreover, all the crispants without indels also showed base changes causing premature stop codons. The control embryos injected with Cas9 mRNA alone did not show any mutations at both the guide RNA loci.

Given the high efficiency the sgRNAs and the generation of indels and premature stop codons in the crispants, we expect that the injection of both the sgRNAs targeting exon 1 would result in effective disruption of fmn2b.

We have now indicated this in the Methods section.

3. The morphological phenotypes for both morphants and crispants only received a score as either having a phenotype or not. Scoring is not a robust way of assessing phenotypes that needs strong controls. In addition, there is no mention of making the scorer unaware of the expected outcome (“blinding”) of the assessment. In combination, scoring and lack of blinding make phenotype quantification open to a very high level of experimental bias. The authors should provide more quantitative analysis of the phenotypes. For example, they could first binarize their images in ImageJ (using a consistent threshold) before analysing the presence or absence of spiral tract or quantifying the signal in another way in a blind manner.

The scoring of the phenotypes was done in a genotype blinded manner. We have now mentioned this in the materials and methods section of the manuscript.

To alleviate the concerns regarding scoring bias, as suggested by the reviewers, we re-analyzed the presence or absence of spiral fiber neurons in morphants and crispants by intensity analysis post uniform thresholding. A straight-line ROI spanning both the spiral fiber tracts (see A in the figure below) was used to plot the fluorescence signal intensity profiles. The plots showing two distinct peaks was scored as having two spiral fiber tracts, whereas, the plots with no distinct peaks were scored as the absence of the spiral fiber tracts. Representative plots of the intensity profiles are shown below (B). The percentage of embryos showing the phenotype remained the same in both the morphants and crispants when compared to the manual analysis.

-

4. Phenotypes are described in the text that are not depicted in the images. For example, a “significant thinning of the tracts” is described, but this is not convincingly shown or quantified in Fig. 5. Moreover, it is stated that in line 452f of the discussion that spiral fibres fail to form synaptic terminals at the M-cell axon hillock. This is not described in the results and not visible in the images.

We have added representative images depicting the thinning of the spiral fiber tract in the extended data figure 5-1 associated with Figure 5. We have reported the proportion of embryos showing discernible thinning. However, we are unable to measure the thickness of the tract reliably from our images and have only scored tracts with visually obvious decrease in the thickness. We are indicating this in Methods section to avoid ambiguity.

We have rephrased the sentence in line 452f of the discussion. The sentence was intended to highlight the consequence of the spiral fiber neuron tract defects described here, wherein the absence of the spiral fiber tracts indicates the absence of the otherwise excitatory feedforward input at the M-cell hillock (Lacoste et al., 2014).

Minor points:

1. As the authors mentioned in their reply, Fmn2 should be written with a first letter upper case and not italicized when describing the protein. Many sentences in the introduction describe the protein and its functions. They should therefore use Fmn2 rather than fmn2, as it was written in the first submission (lines 5, 27, 70-79, etc).

This suggestion has been implemented throughout the text.

2. Typos / wording:

- Line 39: remove “the” between “during” and “development"

- Line 92: remove “the” before “fmn2 expression"

- Line 157: add “of” between “pg” and “both"

- Lines 385: remove “the axonal tracts of” (redundant with “the Spiral fiber tracts”), or replace “the Spiral fiber tracts” by “the Spiral fiber neurons"

The typographical/wording errors have been corrected in the manuscript.

REFERENCES

Lacoste AMB, Schoppik D, Robson DN, Haesemeyer M, Portugues R, Li JM, Randlett O, Wee CL, Engert F, Schier AF (2014) A Convergent and Essential Interneuron Pathway for Mauthner-Cell-Mediated Escapes. Curr Biol:1526-1534.

Lorent K, Liu KS, Fetcho JR, Granato M (2001) The zebrafish space cadet gene controls axonal pathfinding of neurons that modulate fast turning movements. Development 128:2131-2142.
