# Supplementary figures and images for: The Formin Fmn2b Is Required for the Development of an Excitatory Interneuron Module in the Zebrafish Acoustic Startle Circuit
Source: eNeuro. 2021 Jul 8;8(4):ENEURO.0329-20.2021. doi: 10.1523/ENEURO.0329-20.2021 (PMC8272403; doi:10.1523/ENEURO.0329-20.2021)

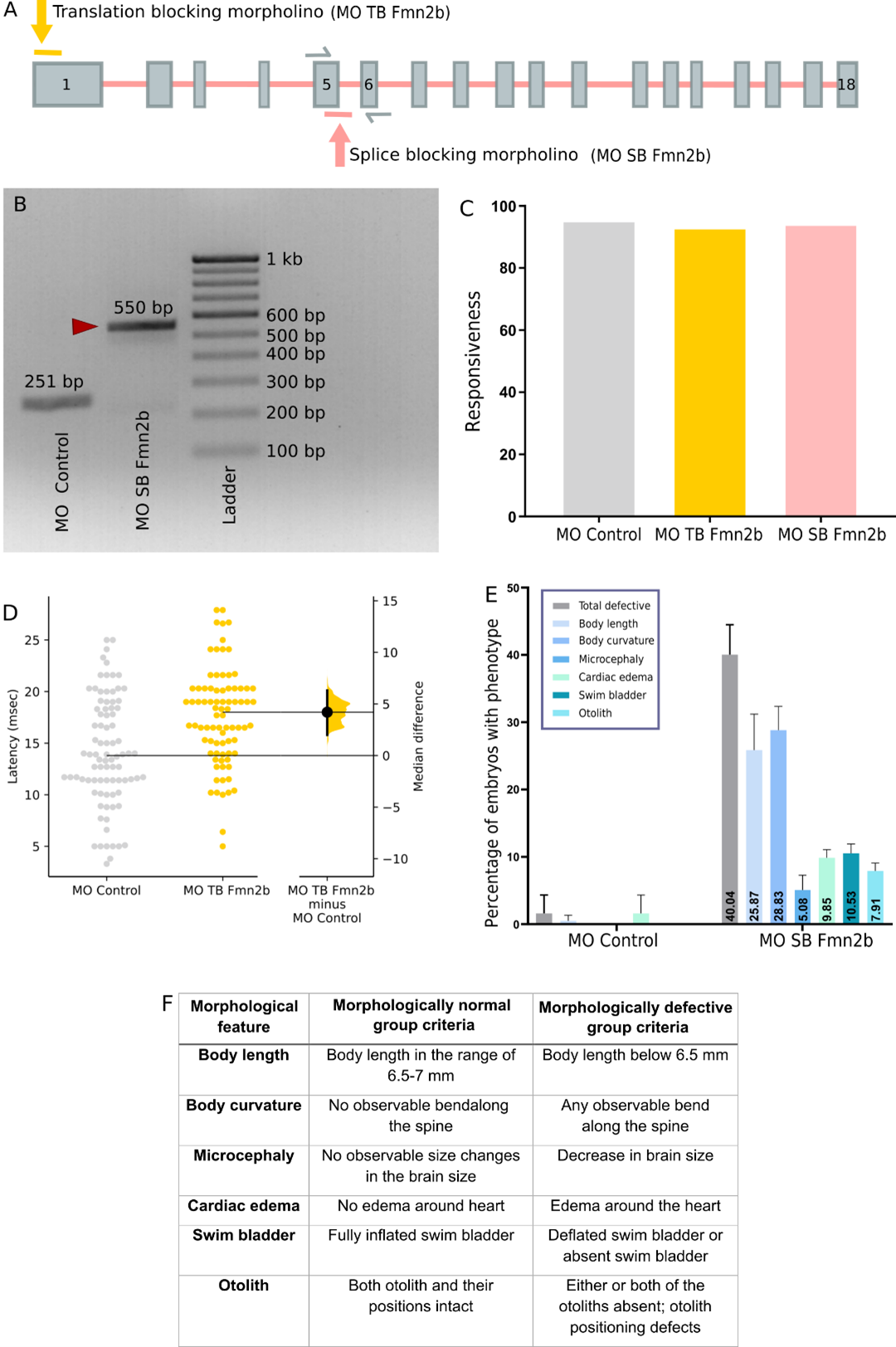

Supplement: Extended Data Figure 2-1 — Morpholino design, validation, and assessment of morphological defects in morphants. A, Schematic showing target regions for the two morpholinos used in the experiments. MO TB Fmn2b targets exon 1 to block translation. MO SB Fmn2b targets the exon 5–intron 5 boundary to cause retention of intron 5 between exons 5 and 6, leading to the occurrence of a premature stop codon. Both morpholinos ensure that the functional domains are not translated in Fmn2b morphants. B, Validation of knock-down by MO SB Fmn2b morpholino was done using RT-PCR on cDNA obtained from MO control and MO SB Fmn2b-injected embryos. The amplification of a 550-bp amplicon from MO SB Fmn2b morphants cDNA corresponds to inclusion of intron 5 because of efficient splice blocking by the morpholino. C, Responsiveness is quantified as the percentage of larvae responding to acoustic stimuli in MO control (95.2%), MO TB Fmn2b (92.9%), and MO SB Fmn2b (94%)-injected embryos. D, Behavioral analysis of MO TB Fmn2b morphants is summarized in the Cumming plot. MO TB Fmn2b morphants also exhibit increased latency defect. The unpaired median difference between MO control and MO TB Fmn2b is 4.2 [95.0%CI 2.0, 6.3]. The p value of the two-sided permutation t test is 0.0004. The effect sizes and CIs are reported as effect size [CI width lower bound; upper bound]. E, Graph summarizing morphological defects in Fmn2b morphants injected with 2 ng of MO SB Fmn2b. The percentage of embryos showing the various defects are indicated at the bottom of each bar corresponding to the defect. A total of 40.04% embryos in the morphant population showed defects spanning from a few or all of the defects listed. Each bar represents the percentage of embryos exhibiting that morphological defect. Morphologically aberrant embryos often exhibited multiple defects together. F, Table enlisting the parameters used to distinguish between morphologically aberrant and normal embryos. These parameters were used for classification of both morphan [file enu-eN-NWR-0329-20-s06.tif]

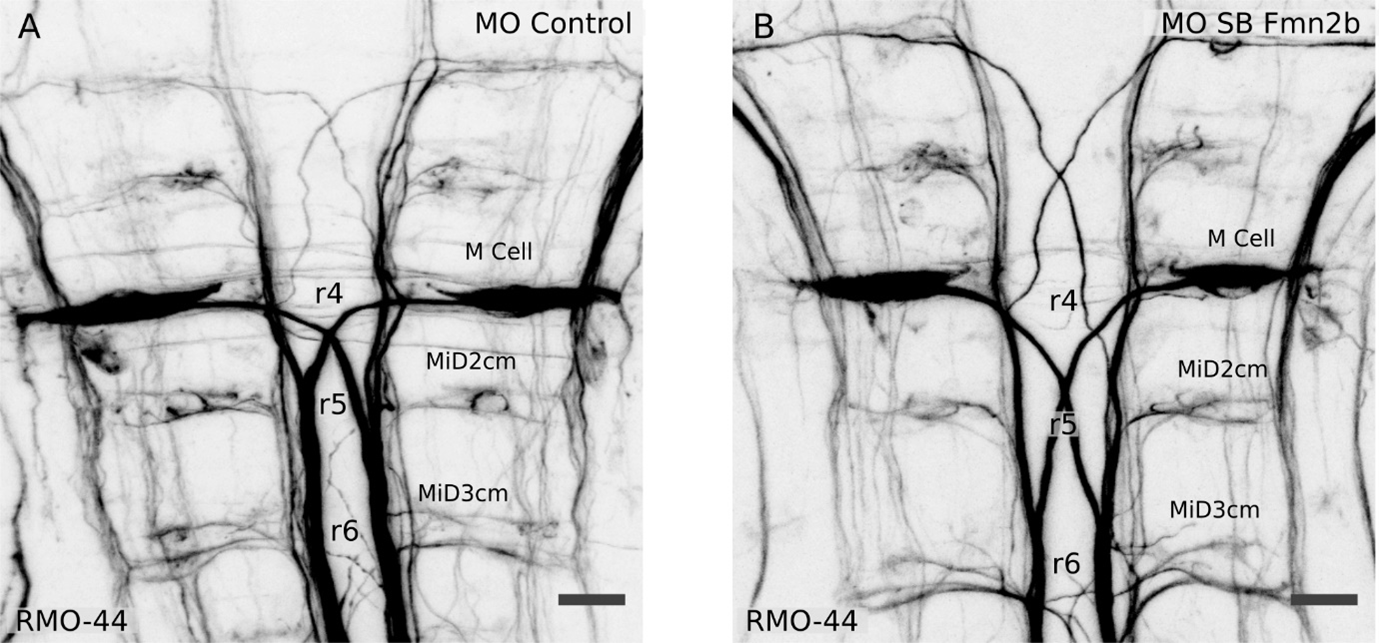

Supplement: Extended Data Figure 4-1 — Reticulospinal neuron cell bodies remain unaffected in Fmn2b morphants. Whole-mount immunostaining using RMO-44 antibody of 48-hpf (A) 2 ng MO control morpholino injected and (B) 2 ng MO SB Fmn2b morpholino-injected embryos shows that there are no significant changes in the cell bodies of reticulospinal neurons at early stages. Scale bar: 20 μm. Download Figure 4-1, TIF file. [file enu-eN-NWR-0329-20-s05.tif]

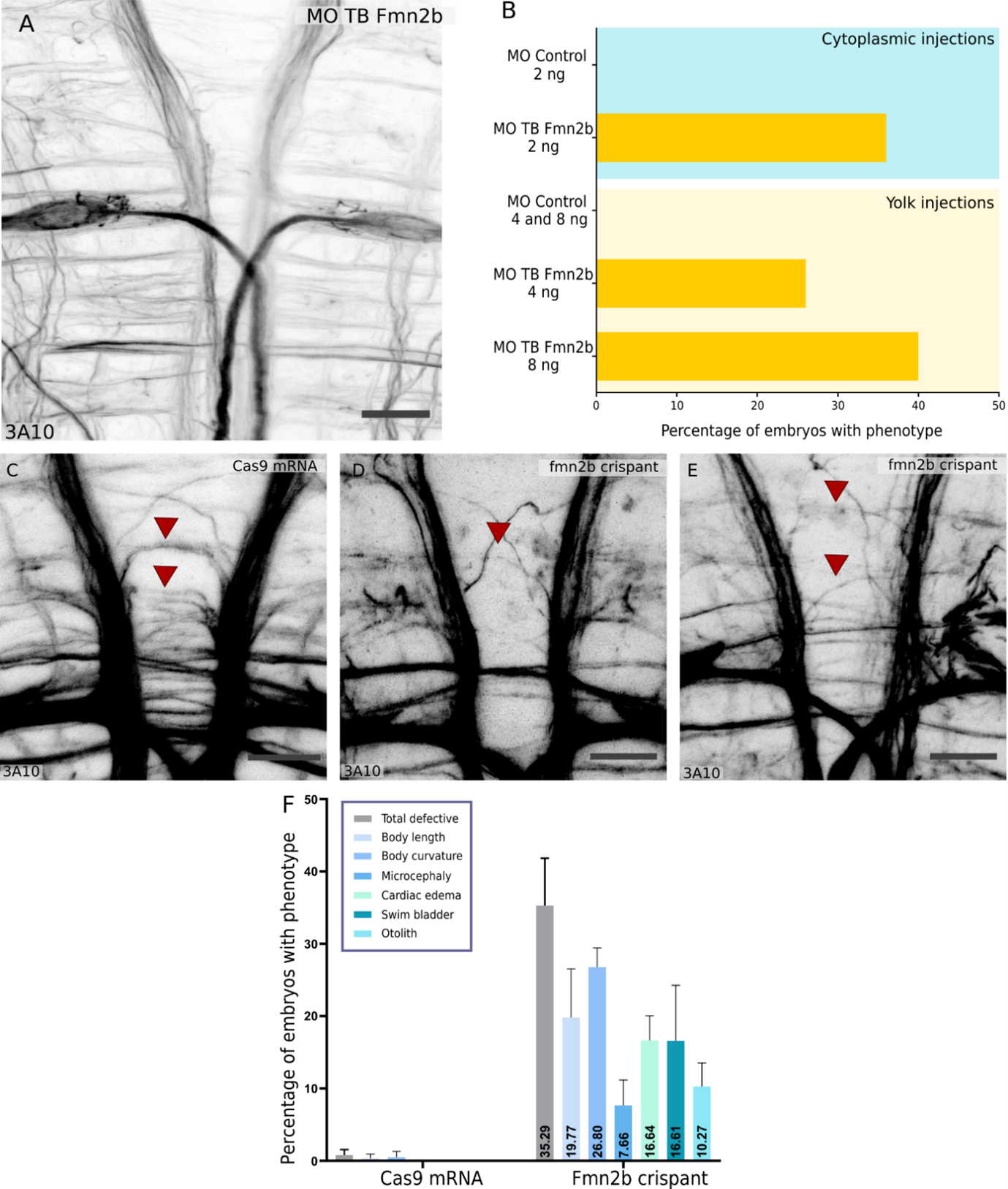

Supplement: Extended Data Figure 5-1 — The translation blocking morpholino recapitulates the spiral fiber neuron defect in Fmn2b morphants. A, Whole-mount immunostaining using 3A10 antibody of 96-hpf Fmn2b morphants injected with 2 ng MO TB Fmn2b phenocopies the splice blocking Fmn2b morphant defects. Scale bar: 20 μm. B, Quantification of cytoplasmic injections of 2 ng MO TB Fmn2b and yolk injections of higher doses (4 and 8 ng) of MO TB Fmn2b. Both cause the spiral fiber neuron outgrowth defect in a dose-dependent manner. Representative micrographs showing the (D) absence of only one tract and (E) thinning of the spiral fiber tract in Fmn2b crispants as compared to (C) embryos injected with only Cas9 mRNA. F, Quantification of morphological defects in Fmn2b crispants injected with 100 pg each of sgRNA1 and sgRNA2 along with 300 pg of Cas9 mRNA at 96 hpf. The parameters used for classification of morphologically aberrant embryos were the same as in Extended Data Figure 2-1F. Download Figure 5-1, TIF file. [file enu-eN-NWR-0329-20-s04.tif]

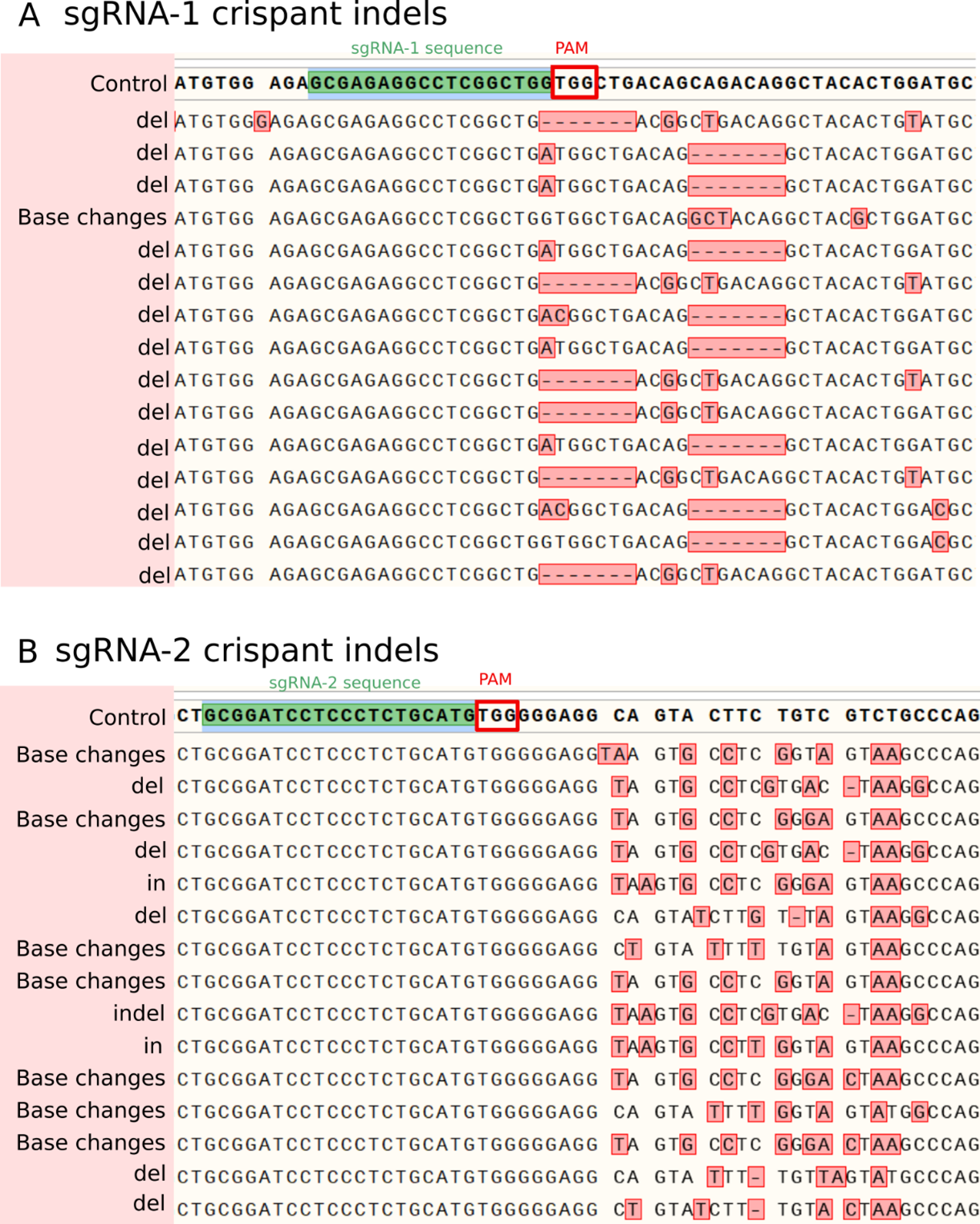

Supplement: Extended Data Figure 5-2 — Summary of indels and base changes generated in Fmn2b crispants. Out of 15 randomly chosen crispants injected with sgRNA-1, sgRNA-2 and Cas9 mRNA, (A) 93.3% embryos exhibited indels at the sgRNA-1 locus, and (B) 53.3% embryos showed indels at the sgRNA-2 locus on exon 1 of fmn2b. Remaining larvae also exhibited base changes at the both the loci. in: insertion; del: deletion. Download Figure 5-2, TIF file. [file enu-eN-NWR-0329-20-s03.tif]
